# GSK3β is a critical, druggable component of the network regulating the active NOTCH1 protein and cell viability in CLL

**DOI:** 10.1038/s41419-022-05178-w

**Published:** 2022-09-01

**Authors:** Filomena De Falco, Chiara Rompietti, Daniele Sorcini, Angela Esposito, Annarita Scialdone, Stefano Baldoni, Beatrice Del Papa, Francesco Maria Adamo, Estevão Carlos Silva Barcelos, Erica Dorillo, Arianna Stella, Mauro Di Ianni, Isabella Screpanti, Paolo Sportoletti, Emanuela Rosati

**Affiliations:** 1 Department of Medicine and Surgery, Institute of Hematology, Centro di Ricerca Emato-Oncologica (CREO), University of Perugia, Perugia, Italy; 2 Department of Medicine and Sciences of Aging, “G. d’Annunzio” University of Chieti-Pescara, Chieti, Italy; 3 Department of Oncology and Hematology, Ospedale Civile “Santo Spirito”, ASL Pescara, Pescara, Italy; 4 Department of Molecular Medicine, University of Rome “La Sapienza”, Rome, Italy; 5 Department of Medicine and Surgery, University of Perugia, Perugia, Italy

**Keywords:** Chronic lymphocytic leukaemia, Target identification

## Abstract

NOTCH1 alterations have been associated with chronic lymphocytic leukemia (CLL), but the molecular mechanisms underlying NOTCH1 activation in CLL cells are not completely understood. Here, we show that GSK3β downregulates the constitutive levels of the active NOTCH1 intracellular domain (N1-ICD) in CLL cells. Indeed, GSK3β silencing by small interfering RNA increases N1-ICD levels, whereas expression of an active GSK3β mutant reduces them. Additionally, the GSK3β inhibitor SB216763 enhances N1-ICD stability at a concentration at which it also increases CLL cell viability. We also show that N1-ICD is physically associated with GSK3β in CLL cells. SB216763 reduces GSK3β/N1-ICD interactions and the levels of ubiquitinated N1-ICD, indicating a reduction in N1-ICD proteasomal degradation when GSK3β is less active. We then modulated the activity of two upstream regulators of GSK3β and examined the impact on N1-ICD levels and CLL cell viability. Specifically, we inhibited AKT that is a negative regulator of GSK3β and is constitutively active in CLL cells. Furthermore, we activated the protein phosphatase 2 A (PP2A) that is a positive regulator of GSK3β, and has an impaired activity in CLL. Results show that either AKT inhibition or PP2A activation reduce N1-ICD expression and CLL cell viability in vitro, through mechanisms mediated by GSK3β activity. Notably, for PP2A activation, we used the highly specific activator DT-061, that also reduces leukemic burden in peripheral blood, spleen and bone marrow in the E*µ*-TCL1 adoptive transfer model of CLL, with a concomitant decrease in N1-ICD expression. Overall, we identify in GSK3β a key component of the network regulating N1-ICD stability in CLL, and in AKT and PP2A new druggable targets for disrupting NOTCH1 signaling with therapeutic potential.

## Introduction

Chronic lymphocytic leukemia (CLL) is a hematological malignancy characterized by accumulation of CD19^+^/CD5^+^ cells resistant to apoptosis due to genetic lesions and microenvironment stimuli [1–3]. Despite advances in treatment, CLL remains an incurable disease, suggesting that a dissection of the pathways supporting CLL cell growth/survival is necessary to identify new therapeutic targets.

Growing evidence link CLL to activated NOTCH1 signaling [4]. CLL cells have a constitutive NOTCH1/2 activation sustaining their apoptosis resistance [5–7]. NOTCH1 is also activated in CLL stem cell compartment [8]. Mutations in *NOTCH1* PEST domain are the most common genetic lesions in CLL with poor outcome affecting up to 20% of patients [9–13]. These mutations increase NOTCH1 signaling because they result in impaired proteasomal degradation of the active NOTCH1 intracellular domain (N1-ICD) [14, 15]. However, NOTCH1 is also activated in unmutated CLL patients who show transcriptional programs similar to those of mutated cases [16], indicating a broader role of NOTCH1 signaling in CLL. These studies along with recent evidence showing that NOTCH1 signaling promotes disease initiation and progression in a mouse model of CLL [17], indicate the need to define the mechanisms sustaining NOTCH1 activation in CLL for the development of NOTCH1-targeted therapies.

Previous studies showed that in CLL cells residing into lymph node or bone marrow niches, NOTCH1 is activated by *trans*-interactions with the NOTCH ligands expressed on neighboring normal cells [14, 18]. In peripheral blood CLL cells, ligand-dependent mechanisms are rather unlikely, as suggested by our previous evidence that NOTCH1/JAGGED1 interactions among CLL cells are not responsible for NOTCH1 activation [19]. Conversely, a role in sustaining the levels of N1-ICD is played by IL-4-induced PI3Kδ/AKT pathway [19] and BCR-induced BTK [20, 21], suggesting that crosstalk among multiple deregulated signaling molecules controls NOTCH1 activation in CLL.

GSK3β is a multi-functional serine/threonine kinase with a crucial role in regulating cell proliferation, differentiation and survival [22–24]. GSK3β activity is regulated by multiple pathways through phosphorylation/dephosphorylation events and other mechanisms, including differential splicing, subcellular localization, interaction with scaffold proteins and proteolytic cleavage [22–24]. Specifically, GSK3β, constitutively active in resting cells, is inactivated through an inhibitory phosphorylation at Serine 9 (S9) by several pathways, including PI3K/AKT, PKA, PKC, p90RSK and p70S6K [25–27], with many of them which are aberrantly activated in CLL cells [2]. Conversely, GSK3β activity can be restored by protein phosphatases, as the serine/threonine phosphatase 2 A (PP2A), whose activity is impaired in several malignancies, including CLL [28–30].

Deregulated GSK3β activity is involved in many malignancies by controlling several oncoproteins, including c-myc, cyclin D1, Mcl-1 and NOTCH receptors [31–33]. The role of GSK3β in NOTCH regulation is controversial because in different cell types, it has been shown that GSK3β can either positively [34–36] or negatively [37–41] regulate NOTCH signaling, both at transcriptional level and in terms of protein stability.

Based on these notions, and as GSK3β represents a crucial converging point for multiple pathways that are deregulated in CLL, it is conceivable that GSK3β is a player of the network regulating NOTCH1 activation in CLL with potential for drug targeting.

Here, we showed that GSK3β downregulates N1-ICD levels in CLL cells and plays an antileukemic role in CLL. Furthermore, we identified in the upstream regulators of GSK3β, AKT and PP2A, new druggable targets for reducing NOTCH1 signaling and CLL cell survival.

## Results

### GSK3β modulation regulates N1-ICD levels in CLL cells

**Fig. 1:**
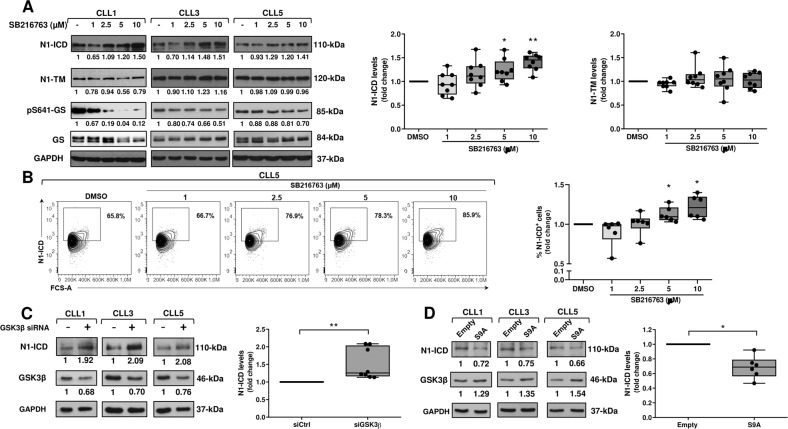
GSK3β modulation regulates N1-ICD levels in CLL cells. **A**, **B** Primary CLL cells were cultured for 3 h with the indicated concentrations of SB216763 or DMSO as control. **A** Western blot analysis of NOTCH1 was performed using the anti-NOTCH1 (Val1744) and the anti-NOTCH1 (D1E11) antibodies, able to detect N1-ICD and N1-TM, respectively (*n* = 8). Protein loading was assessed using an anti-GAPDH antibody. Left, the values under each blot indicate the fold change in N1-ICD and N1-TM expression in SB216763-treated cells compared with control DMSO (set to 1), normalized to GAPDH levels. GSK3β activity inhibition by SB216763 was assessed by analyzing the glycogen synthase phosphorylation at Serine 641 (pS641-GS). The values under each blot indicate the fold change in pS641-GS levels in SB216763-treated cells compared with control DMSO (set to 1), normalized to levels of total GS. Three CLL samples are shown. Right, box and whisker plots with data points of densitometry analysis of N1-ICD and N1-TM, represented as fold change compared with control DMSO. *P < 0.05, **P < 0.01 according to Wilcoxon paired test. **B** Flow cytometric analysis of N1-ICD performed using the mouse anti-NOTCH1 (mN1A)-PE antibody (*n* = 6). Left, results are represented as the percentage of N1-ICD positive cells. N1-ICD positive gate was set based on staining with PE-mouse IgG isotype control. One CLL sample is shown. Right, box and whisker plots with data points of the percentage of N1-ICD positive cells, represented as fold change compared with control DMSO set to 1. *P < 0.05 according to Wilcoxon paired test. **C** CLL cells were transfected with control siRNA (siCtrl) or GSK3β siRNA (siGSK3β) (*n* = 8). Left, N1-ICD expression was analyzed as in panel A. Protein loading was assessed using an anti-GAPDH antibody. Silencing efficiency was assessed by Western blot analysis of GSK3β. The values under each blot indicate the fold change in N1-ICD and GSK3β levels in siGSK3β cells compared with siCtrl cells (set to 1), normalized to GAPDH levels. Three CLL samples are shown. Right, box and whisker plots with data points of densitometry analysis of N1-ICD, represented as fold change compared with siCtrl cells. **P < 0.01 according to Wilcoxon paired test. **D** CLL cells were transiently transfected with the pcDNA3.1 empty vector as control or the pcDNA3 plasmid containing the constitutively active GSK3β (GSK3β S9A) (*n* = 6). Left, N1-ICD expression was analyzed as in panel A. Protein loading was assessed using an anti*-*GAPDH antibody. Transfection efficiency was assessed by Western blot analysis of GSK3β. The values under each blot indicate the fold change in N1-ICD and GSK3β expression in S9A-transfected cells compared with empty vector-transfected cells (set to 1), normalized to GAPDH levels. Three CLL samples are shown. Right, box and whisker plots with data points of densitometry analysis of N1-ICD, represented as fold change compared with empty vector-transfected cells. *P < 0.05 according to Wilcoxon paired test.

All experiments of this study were performed in CLL cells isolated from *NOTCH1* wild-type patients. To determine whether GSK3β plays a role in regulating NOTCH1 signaling in CLL cells, we examined the effects of GSK3β activity modulation on constitutive NOTCH1 protein. We first inhibited GSK3β activity by culturing CLL cells for 3 h with increasing concentrations of the GSK3β inhibitor SB216763 [42] or DMSO as control (*n* = 8), before Western blot (WB) analysis of the active NOTCH1 intracellular domain (N1-ICD) and NOTCH1-transmembrane subunit (N1-TM). Figure 1A shows that SB216763 increased the constitutive levels of N1-ICD, with 5 and 10 μM which induced the highest effects, whereas N1-TM levels remained unchanged at all doses analyzed. The effect of SB216763 on N1-ICD was rapid, as the increase was observed already after 1.5 h-treatment (*n* = 3; Supplementary Fig. S1). GSK3β inhibition by SB216763 was confirmed by reduced phosphorylation at Serine 641 of glycogen synthase (pS641-GS), a GSK3β specific substrate (Fig. 1A and Supplementary Fig. S1). We then performed flow cytometric analysis of N1-ICD in CLL cells treated for 3 h with the same concentrations of SB216763 used for WB experiments (*n* = 6). The data shown in Fig. 1B are consistent with those obtained by WB analysis.

To better investigate the role of GSK3β in N1-ICD control, we downregulated GSK3β expression by using small interfering RNA (siRNA). CLL cells were transfected with control nontargeting (siCtrl) or specific GSK3β siRNA (siGSK3β), cultured for 48 h in complete medium, and analyzed for GSK3β and N1-ICD proteins (*n* = 8). Figure 1C shows that reduction in GSK3β expression, induced by siGSK3β transfection compared with siCtrl cells, was accompanied by increased N1-ICD levels, suggesting, in keeping with pharmacologic inhibition studies, that GSK3β is a negative regulator of N1-ICD protein in CLL cells.

To definitively establish the role of GSK3β in N1-ICD regulation, we analyzed N1-ICD expression in CLL cells (*n* = 6) transiently transfected with an empty plasmid as control, or a plasmid containing a constitutively active GSK3β, which has the Serine 9 (S9) mutated to Alanine (S9A) and cannot be phosphorylated, remaining in an activated status [43]. S9A-GSK3β transfection in CLL cells was demonstrated by the increased GSK3β expression. Results showed that after 48 h-transfection, N1-ICD levels were reduced in S9A-GSK3β transfected compared with control cells, confirming that GSK3β negatively regulates N1-ICD levels in CLL cells (Fig. 1D).

### Pharmacologic GSK3β inhibition enhances N1-ICD stability and CLL cell viability

**Fig. 2:**
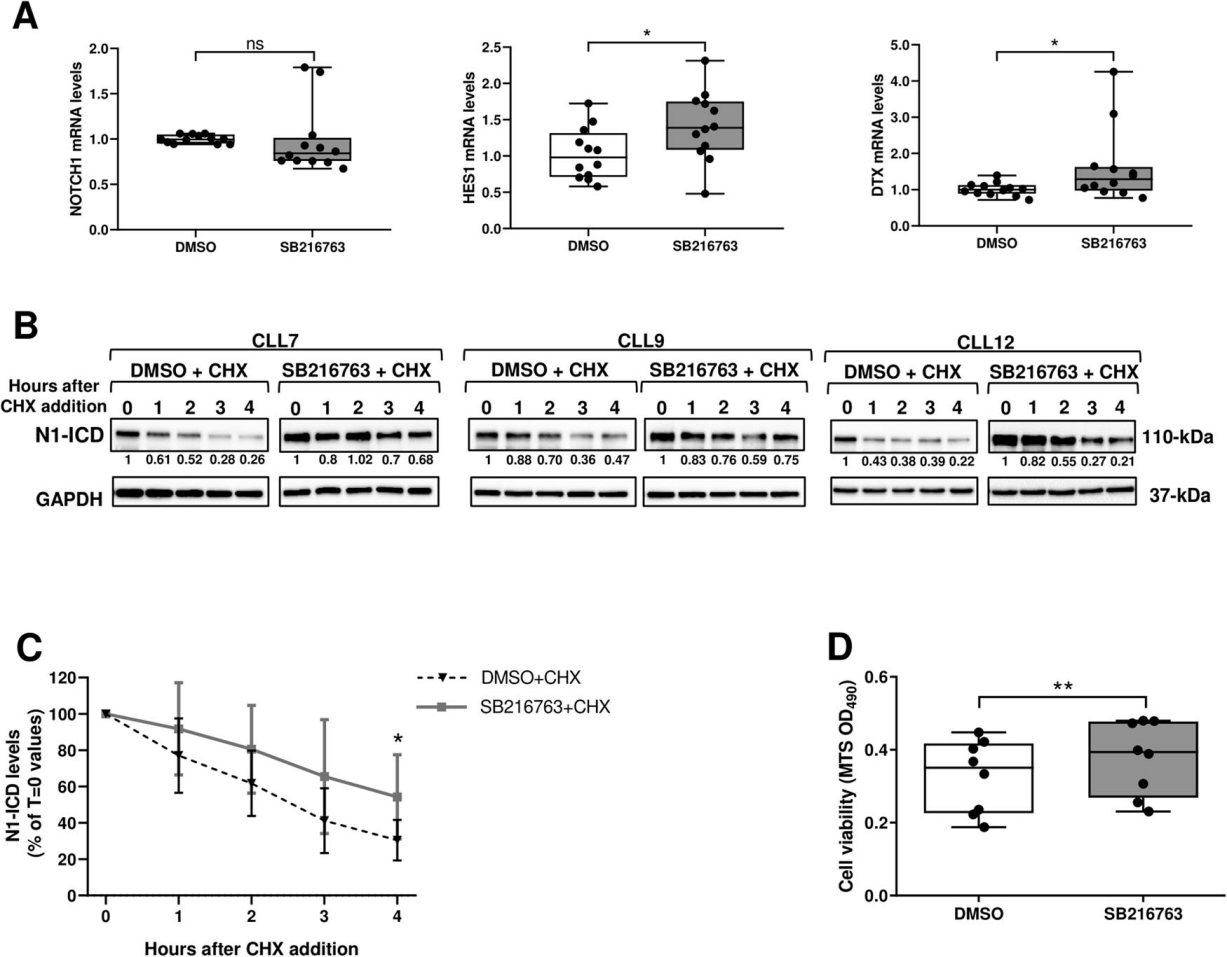
Pharmacologic GSK3β inhibition enhances N1-ICD stability and CLL cell viability. **A** Box and whisker plots with data points of real-time PCR analysis of NOTCH1, HES1 and DELTEX (DTX) mRNA in CLL cells cultured for 3 h with 5 μM SB216763 or DMSO as control (*n* = 12). mRNA levels were normalized to GAPDH and represented as fold change using control cells as a reference. *P < 0.05; ns, not significant, according to Wilcoxon paired test. **B**, **C** After pretreatment with 5 μM SB216763 or DMSO for 1.5 h, CLL cells were treated (*T* = 0) with 50 μg/ml CHX and harvested at the indicated times for Western blot analysis of N1-ICD and GAPDH, as a loading control (*n* = 6). **B** The values under the blots relative to each treatment indicate the fold change in N1-ICD expression at the different time points compared with the respective *T* = 0 (set to 1), normalized to GAPDH levels. Three CLL samples are shown. **C** N1-ICD bands were quantified by densitometry analysis, normalized to GAPDH and represented as percentage of *T* = 0 value set to 100%. Data are presented as the mean ± SD of 6 CLL samples. *P < 0.05 according to Wilcoxon paired test. **D** CLL cells were cultured for 18 h with 5 μM SB216763 or DMSO as control (*n* = 8). Cell viability was measured by MTS assay. Box and whisker plots with data points, expressed as optical density (OD) values, are shown. **P < 0.01 according to Wilcoxon paired test.

To elucidate the mechanisms by which GSK3β downregulates N1-ICD levels in CLL cells, we examined the effect of GSK3β pharmacologic inhibition on transcriptional expression of NOTCH1 and its downstream targets HES1 and DELTEX (DTX) (*n* = 12). Figure 2A shows that SB216763 treatment (5 µM for 3 h) increased HES1 and DTX mRNA compared with controls, but did not influence NOTCH1 mRNA levels, suggesting that GSK3β downregulates N1-ICD by posttranscriptional mechanisms. Then, we determined the effect of SB216763 on N1-ICD stability and assessed N1-ICD levels in CLL cells pretreated with SB216763 or DMSO for 1.5 h, and then with the translational inhibitor cycloheximide (CHX; 50 µg/ml) for different times (*n* = 6). Figure 2B and C and Supplementary Fig. S2 show that pretreatment with SB216763 attenuated the decrease of N1-ICD observed in cells treated with CHX alone. These data indicate that GSK3β inhibition enhances N1-ICD stability, suggesting that GSK3β is involved in N1-ICD degradation.

Given that inhibition of GSK3β enhances NOTCH1 signaling, which has been shown to promote CLL cell survival [5–7], we performed MTS assay in CLL cells treated with SB216763 for 18 h (*n* = 8), to assess whether GSK3β directly modulates CLL cell viability. Consistent with previous studies [44], SB216763 increased CLL cell viability compared with controls, indicating that GSK3β plays an antileukemic role in CLL (Fig. 2D).

### GSK3β interacts with N1-ICD in CLL cells

**Fig. 3:**
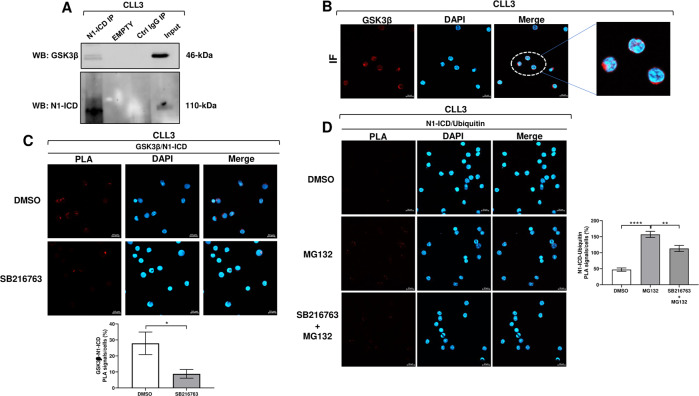
GSK3β interacts with N1-ICD in CLL cells and is involved in N1-ICD ubiquitination. **A** N1-ICD was immunoprecipitated (IP) from whole-cell extracts of CLL cells, and the IP lysates were analyzed by Western blot with the anti-NOTCH1 (Val1744) to confirm IP of N1-ICD, and with the anti-GSK3β antibody to detect GSK3β/N1-ICD interaction (*n* = 3). One representative CLL is shown. **B** Confocal microscopy images of subcellular localization of GSK3β in a representative CLL sample. CLL cells (*n* = 3) were stained with the anti-GSK3β antibody (red) and with DAPI for nuclei (blue) and then analyzed by confocal microscopy, with a 63x oil immersion and 1.4 NA objective; scale bar, 10 μm. **C**, **D** PLA was performed by using rabbit anti-NOTCH1 (Val1744) and mouse anti-GSK3β antibodies to detect GSK3β/N1-ICD interactions in CLL cells cultured for 1.5 h with 5 µM SB216763 or DMSO (**C**; *n* = 3), and by using rabbit anti-NOTCH1 (Val1744) and mouse anti-ubiquitin antibodies to detect N1-ICD/Ubiquitin interactions in CLL cells cultured with 5 µM SB216763 or DMSO for 1.5 h, and with 10 µM MG132 for additional 4 h (**D**; *n* = 3). Nuclei were stained with DAPI. In the confocal microscopy images, red spots indicate GSK3β/N1-ICD (**C**) and N1-ICD/Ubiquitin (**D**) interactions. Images were acquired by using confocal microscopy with a 63x oil immersion and 1.4 NA objective; scale bar, 10 μm. One representative CLL is shown. In the bottom panel (**C**) and in the right panel (**D**), bar graphs ± SEM show quantitative analysis of the PLA signals of three samples. ****P < 0.0001; **P < 0.01; *P < 0.05 according to unpaired Student’s *t-*test.

In an attempt to understand if GSK3β could interact with N1-ICD in CLL cells, first, we immunoprecipitated N1-ICD protein from CLL cells and analyzed the lysates with an anti-GSK3β antibody (*n* = 3). Results showed the presence of immunocomplexes containing both GSK3β and N1-ICD, suggesting a possible interaction between the two proteins (Fig. 3A).

Then, we performed confocal immunofluorescence analysis to define the subcellular localization of GSK3β in CLL cells, and showed that GSK3β resided mainly in the cytoplasm (*n* = 3; Fig. 3B). To better investigate GSK3β/N1-ICD interactions and to define whether pharmacologic GSK3β inhibition influenced them, we performed proximity ligation assay (PLA) in CLL cells cultured for 1.5 h with 5 μM SB216763 or DMSO (*n* = 3). Results revealed the presence of PLA red spots in control cells, indicating a close proximity between N1-ICD and GSK3β (Fig. 3C). SB216763 induced a decrease in the percentage of PLA signals, suggesting that the proximity between N1-ICD and GSK3β is disadvantaged when GSK3β is less active (Fig. 3C), condition that is associated with increased N1-ICD stability.

### GSK3β is involved in N1-ICD ubiquitination in CLL cells

We showed that N1-ICD is degraded by proteasome in CLL cells. N1-ICD levels declined after 5 h-culture compared with freshly-isolated cells, but recovered in the presence of the proteasome inhibitor MG132 (*n* = 3; Supplementary Fig. S3). Based on this observation and since SB216763 reduced GSK3β/N1-ICD interactions while increased N1-ICD stability in CLL cells, we hypothesized that GSK3β/N1-ICD interactions might be important for targeting N1-ICD towards the ubiquitin-proteasome pathway. We thus determined whether GSK3β inhibition by SB216763 affected the amount of ubiquitinated N1-ICD. We treated CLL cells with 5 µM SB216763 or DMSO for 1.5 h and with 10 µM MG132 for additional 4 h, and then we examined N1-ICD/ubiquitin complexes by PLA, using anti-N1-ICD and anti-ubiquitin antibodies (*n* = 3). Results showed that PLA signals were increased in CLL cells treated with MG132 compared with DMSO (Fig. 3D), indicating an accumulation of N1-ICD/ubiquitin complexes. Strikingly, PLA signals were reduced in CLL cells treated with SB216763 and MG132 compared with cells treated with MG132 alone (Fig. 3D), indicating that lower amounts of N1-ICD undergo ubiquitin-proteasome pathway when GSK3β is less active. These data along with the above results showing that GSK3β inhibition enhances N1-ICD stability further indicate that an active GSK3β promotes N1-ICD proteasomal degradation.

### High N1-ICD levels correlate with GSK3β inactivation in CLL cells

**Fig. 4:**
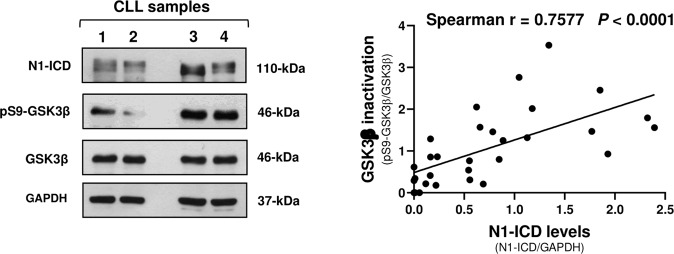
Analysis of the correlation between N1-ICD and GSK3β inactivation levels in CLL cells. Left, the expression of N1-ICD, pS9-GSK3β used as a marker of GSK3β inactivation status, total GSK3β, and GAPDH used as a loading control, was examined by Western blot analysis (*n* = 30). Four representative samples are shown. Right, quantification of N1-ICD bands, normalized to GAPDH levels, and of pS9-GSK3β bands normalized to total GSK3β levels, was performed by densitometric analysis. Correlation between N1-ICD and pS9-GSK3β expression values was assessed by using the Spearman’s correlation coefficient (*r*). *P* < 0.0001.

Based on the above results showing that GSK3β activity downregulates N1-ICD levels in CLL cells, we hypothesized that an inactive GSK3β contributed to sustain N1-ICD levels. The hypothesis that GSK3β is inactive in CLL cells is supported by the evidence that several kinases which inhibit GSK3β activity by S9-phosphorylation [25, 26], as PI3K/AKT, PKA, PKC, p90RSK and p70S6K [22–27], are aberrantly activated by microenvironment in CLL [2, 45]. Thus, we analyzed the basal levels of N1-ICD and pS9-GSK3β, as a marker of GSK3β inactivation, in 30 CLL samples, and investigated whether there was a correlation between pS9-GSK3β levels and those of N1-ICD. Results showed that in CLL cells, N1-ICD levels were positively correlated with those of pS9-GSK3β (*r* = 0.7577), suggesting that N1-ICD levels depend on degree of GSK3β inactivation (Fig. 4).

### Pharmacologic AKT inhibition reduces N1-ICD levels and CLL cell viability by promoting GSK3β activity

A better understanding of the network sustaining N1-ICD levels in CLL cells might identify novel targets for NOTCH1 signaling inhibition, with potential antileukemic effects. Thus, we investigated whether the modulation of some upstream regulators of GSK3β might reduce N1-ICD levels and CLL cell viability.

**Fig. 5:**
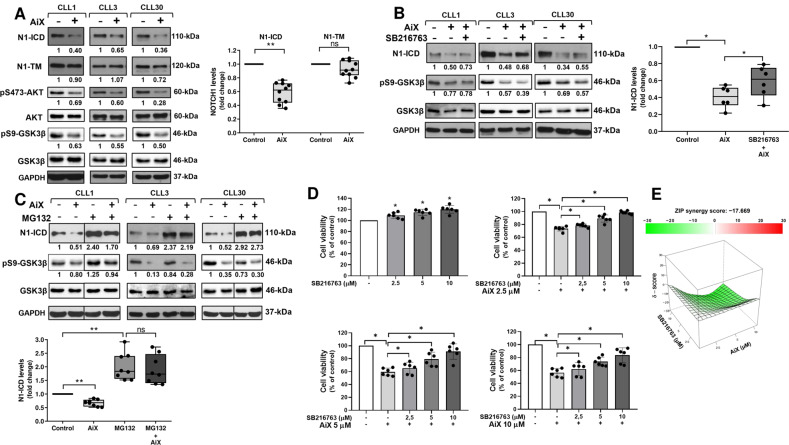
Pharmacologic AKT inhibition reduces N1-ICD levels and CLL cell viability by promoting GSK3β activity. **A** CLL cells were cultured for 6 h with 5 μM AKTiX (AiX) or complete medium as control (*n* = 10). Western blot analysis of NOTCH1 was performed using the anti-NOTCH1 (Val1744) and the anti-NOTCH1 (D1E11) antibodies, able to recognize N1-ICD and N1-TM, respectively. Protein loading was assessed using an anti-GAPDH antibody. Left, the values under each blot indicate the fold change in N1-ICD and N1-TM levels in AiX-treated cells compared with control cells (set to 1), normalized to GAPDH levels. AKT Inhibition by AiX was verified by analyzing AKT phosphorylation at Serine 473 (pS473-AKT). The effect of AiX on GSK3β inactivation was assessed by analyzing pS9-GSK3β levels. The values under each blot indicate the fold change in pS473-AKT and pS9-GSK3β levels in AiX-treated cells compared with control cells (set to 1), normalized to levels of total AKT and total GSK3β, respectively. Three CLL samples are shown. Right, box and whisker plots with data points of densitometry analysis of N1-ICD and N1-TM, represented as fold change compared with controls. **P < 0.01; ns, not significant according to Wilcoxon paired test. **B** CLL cells were cultured for 1.5 h with 5 µM SB216763 or DMSO and for further 6 h with 5 µM AiX (*n* = 6). Western blot analysis of N1-ICD, pS9-GSK3β, total GSK3β and GAPDH was performed as in panel A. Left, the values under the blots indicate the fold change in N1-ICD and pS9-GSK3β levels in cells treated with AiX alone or AiX plus SB216763, compared with control cells (set to 1), normalized to levels of GAPDH and total GSK3β, respectively. Three CLL samples are shown. Right, box and whisker plots with data points of densitometry analysis of N1-ICD, represented as fold change compared with control. *P < 0.05 according to Wilcoxon paired test. **C** CLL cells were pretreated for 2 h with 10 μM MG132 or DMSO, and then cultured for further 6 h with or without 5 μM AiX (*n* = 8). Western blot analysis of N1-ICD, pS9-GSK3β, total GSK3β and GAPDH was performed as in panel A. Top, the values under the blots indicate the fold change in N1-ICD and pS9-GSK3β levels in cells treated with AiX, MG132, or AiX plus MG132, compared with control cells (set to 1), normalized to levels of GAPDH and total GSK3β, respectively. Three CLL samples are shown. Vertical lines inserted in CLL1 and CLL30 blots indicate repositioned gel lanes. Bottom, box and whisker plots with data points of densitometry analysis of N1-ICD, represented as fold change compared with control. **P < 0.01; ns, not significant according to Wilcoxon paired test. **D**, **E** CLL cells were cultured with or without different concentrations of AiX (2.5, 5 and 10 µM) or SB216763 (2.5, 5 and 10 µM) alone or in combinations (*n* = 6). After 18 h, cell viability was measured by MTS assay. **D** Bar graphs with data points of cell viability (mean ± SD) in treated cells compared with untreated controls, set to 100%. *P < 0.05 according to Wilcoxon paired test. **E** The antagonism between SB216763 and AiX was calculated by using the SynergyFinder web application and the results were produced with ZIP Synergy model (green indicates an antagonistic effect, white an additive effect, and red a synergistic effect).

One of the negative regulators of GSK3β is AKT [46], which has been shown to be active in CLL cells [47, 48], and to inactivate GSK3β by S9 phosphorylation in other cell types [25]. Based on these observations and the above results indicating that GSK3β inactivation sustains N1-ICD levels in CLL cells, we investigated whether AKT inhibition affected N1-ICD levels by activating GSK3β. We first analyzed the effect of the AKT inhibitor X (AKTiX, 5 μM for 6 h) on N1-ICD and pS9-GSK3β levels (*n* = 10). Results showed a reduction in N1-ICD with a concomitant decrease in pS9-GSK3β levels, that indicates an increased GSK3β activity (Fig. 5A). AKTiX only marginally affected N1-TM levels (Fig. 5A). Similar effects on N1-ICD and N1-TM were observed in CLL cells treated with the PI3Kδ inhibitor CAL-101 (idelalisib; *n* = 6; Supplementary Fig. S4), suggesting, in agreement with recent studies [49], that the PI3K/AKT pathway plays a critical role in sustaining N1-ICD in CLL. Then, to clarify whether AKT inhibition reduced N1-ICD by activating GSK3β, we examined whether the effect of AKTiX on N1-ICD was affected by the GSK3β inhibitor SB216763. Results showed that N1-ICD levels partially recovered in CLL cells pretreated with SB216763 and then with AKTiX, compared with cells treated with AKTiX alone (*n* = 6; Fig. 5B), suggesting that reduction in N1-ICD levels by AKT inhibition is mediated by GSK3β activity.

Further analysis of the mechanisms by which AKT inhibition downregulates N1-ICD showed that this effect was due to proteasomal degradation, as AKTiX failed to significantly reduce N1-ICD in cells pretreated with MG132 (*n* = 8; Fig. 5C).

Next, given that AKT inhibition reduces CLL cell viability [47, 50], we examined whether this effect was mediated by GSK3β activity. We performed MTS assay in CLL cells cultured for 18 h in the absence or presence of increasing concentrations of AKTiX or SB216763 alone or in combination (*n* = 6). AKTiX decreased CLL cell viability compared with controls at all concentrations used (Fig. 5D), whereas SB216763 increased it (Figs. 5D and 2D). However, the effect of each concentration of AKTiX was antagonized by SB216763 in a dose-dependent manner (Fig. 5D).

The antagonistic effect of SB216763 on AKTiX was confirmed by synergism/antagonism analysis using the SynergyFinder web application [51], which provided a combination score between AKTiX and SB216763 of −17.669 (Fig. 5E). These data indicate that the antileukemic activity induced by AKT inhibition is mediated by GSK3β activity.

Finally, based on the above results showing that AKT contributes to maintain constitutive N1-ICD levels, and other evidence that N1-ICD levels are increased by BCR activation in CLL cells [20, 21], we hypothesized that AKT also contributed to BCR-induced N1-ICD levels. At support of this hypothesis, we found that in CLL cells stimulated for 15 min with 10 µg/ml anti-IgM/IgG antibodies, the increase in N1-ICD levels, induced by BCR stimulation, was prevented by pretreatment with 5 µM AKTiX for 6 h (*n* = 6; Supplementary Fig. S5).

### The PP2A activator DT-061 reduces N1-ICD levels and CLL cell survival in vitro by promoting GSK3β activity

One of the positive regulators of GSK3β is PP2A which activates GSK3β by S9 dephosphorylation [28]. PP2A is a tumor suppressor with reduced activity in CLL and other malignancies [28–30]. This loss-of-function causes several oncogenic events, including dysregulated activity of kinases and other mediators of cancer-cell survival/proliferation, indicating a need for its therapeutic reactivation [28]. There is evidence that in CLL, some indirect PP2A activators exert antileukemic activity abrogating multiple oncogenic signals [52, 53].

**Fig. 6:**
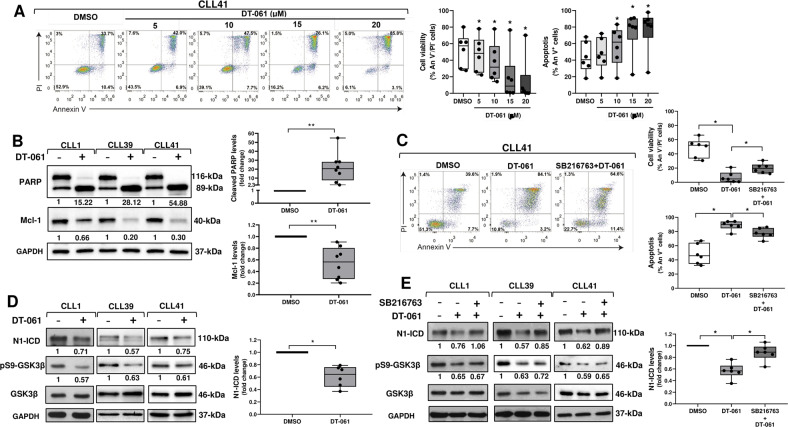
The PP2A activator DT-061 reduces N1-ICD levels and CLL cell survival in vitro by promoting GSK3β activity. **A** CLL cells were treated for 24 h with the indicated concentrations of DT-061 or DMSO as control (*n* = 6). Cell viability and apoptosis were evaluated by flow cytometric analysis of Annexin V/PI (An V/PI) double staining. Left, results are represented as the percentage of viable (An V^-^/PI^-^), early apoptotic (An V^+^/PI^-^), late apoptotic (An V^+^/PI^+^), and necrotic (An V^-^/PI^+^) cells. One CLL sample is shown. Middle and right, box and whisker plots with data points of the percentage of viable An V^-^/PI^-^ (middle) and apoptotic An V^+^ (An V^+^/PI^-^ plus An V^+^/PI^+^) cells (right) are shown. *P < 0.05 according to Wilcoxon paired test. **B** Western blot analysis of PARP and Mcl-1 was performed in CLL cells cultured for 24 h with 15 μM DT-061 or DMSO as control (*n* = 8). GAPDH was analyzed as loading control. Left, the values under the blots indicate the fold change in cleaved PARP (89-kDa) and Mcl-1 levels in DT-061-treated cells compared with control DMSO (set to 1), normalized to levels of full length PARP (116-kDa) and GAPDH, respectively. Three CLL samples are shown. Right, box and whisker plots with data points of densitometry analysis of cleaved PARP (top panel) and Mcl-1 (bottom panel), represented as fold change compared with control DMSO. **P < 0.01 according to Wilcoxon paired test. **C** CLL cells were cultured for 24 h with 15 µM DT-061 as single agent and in combination with 5 µM SB216763, or with DMSO as control (*n* = 6). Cell viability and apoptosis data were obtained and represented as in panel A. Left, one CLL sample is shown. Right, box and whisker plots with data points of the percentage of viable An V^-^/PI^-^ (top panel) and apoptotic An V^+^ (An V^+^/PI^-^ plus An V^+^/PI^+^) cells (bottom panel) are shown. *P < 0.05 according to Wilcoxon paired test. **D** Western blot analysis of N1-ICD was performed in CLL cells cultured for 3 h with 15 μM DT-061 or DMSO as control (*n* = 6). GAPDH was analyzed as loading control. The effect of DT-061 on GSK3β inactivation was assessed by analyzing pS9-GSK3β levels. Left, the values under the blots indicate the fold change in N1-ICD and pS9-GSK3β levels in cells treated with DT-061 compared with control DMSO (set to 1), normalized to levels of GAPDH and total GSK3β, respectively. Three CLL samples are shown. Right, box and whisker plots with data points of densitometry analysis of N1-ICD, represented as fold change compared with control DMSO. *P < 0.05 according to Wilcoxon paired test. **E** Western blot analysis of N1-ICD and pS9-GSK3β was performed in CLL cells pretreated for 1.5 h with 5 μM SB216763 or DMSO, and then cultured for further 3 h with 15 μM DT-061 (*n* = 6). GAPDH was analyzed as loading control. Left, the values under the blots indicate the fold change in N1-ICD and pS9-GSK3β levels in cells treated with DT-061 alone or DT-061 plus SB216763, compared with control DMSO (set to 1), normalized to levels of GAPDH and total GSK3β, respectively. Three CLL samples are shown. Right, box and whisker plots with data points of densitometry analysis of N1-ICD, represented as fold change compared with control DMSO. *P < 0.05 according to Wilcoxon paired test.

Here, to activate PP2A and restrain GSK3β inactivation, we used DT-061, a tricyclic neuroleptic derivative which directly binds and activates PP2A [54], and with a preclinical activity against different tumors [55–58]. We treated CLL cells with increasing concentrations of DT-061 or DMSO as control for 24 h, and then we examined cell viability/apoptosis by flow cytometry after Annexin V/PI (An V/PI) double staining (*n* = 6). Results showed that DT-061 reduced the percentage of viable (An V^-^/PI^-^) CLL cells in a dose-dependent manner, with 15 and 20 μM which induced the highest effects (Fig. 6A, left and middle panels). The reduced cell viability induced by DT-061 was due to apoptosis, as indicated by the increased percentage of An V^+^ cells (An V^+^/PI^-^ plus An V^+^/PI^+^) (Fig. 6A, left and right panels). The apoptotic effect of DT-061 on CLL cells was also supported by the increased cleavage of PARP and the reduced expression of the antiapoptotic Mcl-1 protein observed in DT-061-treated cells compared with controls (*n* = 8; Fig. 6B). To assess whether GSK3β activity mediated the apoptotic effect of DT-061, we analyzed CLL cell viability/apoptosis after 24 h-treatment with 15 μM DT-061 combined with 5 μM SB216763 (*n* = 6). Results showed that combination with SB216763 attenuated the reduction in cell viability as well as the increase in the percentage of An V^+^ cells (An V^+^/PI^-^ plus An V^+^/PI^+^) induced by DT-061 (Fig. 6C, left and right panels), suggesting that in part, GSK3β mediates DT-061 effects.

Next, we investigated the effect of 15 μM DT-061 for 3 h on N1-ICD levels (*n* = 6). Figure 6D shows that DT-061 reduced N1-ICD levels compared with controls and that these effects were accompanied by decreased pS9-GSK3β levels. The reduction in N1-ICD levels induced by DT-061 was mediated by GSK3β activity, because in cells treated for 1.5 h with 5 μM SB216763, and for additional 3 h with 15 μM DT-061, N1-ICD levels were restored compared with cells treated with only DT-061 (Fig. 6E).

### DT-061 exerts antileukemic activity and reduces N1-ICD expression in the E*µ*-TCL1 mouse model of CLL

**Fig. 7:**
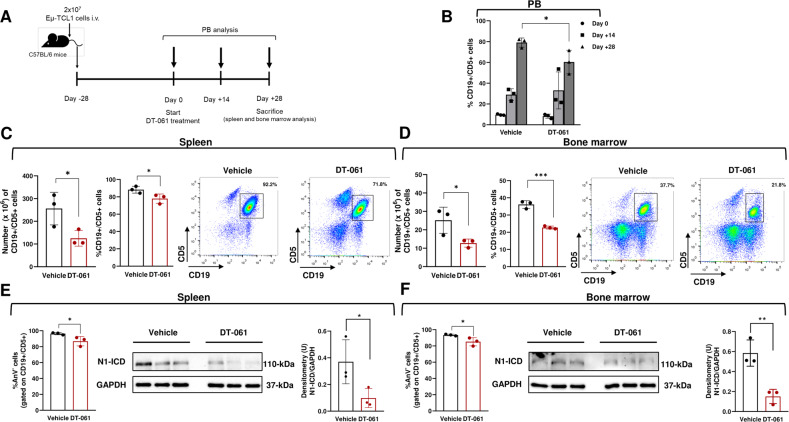
DT-061 exerts antileukemic activity and reduces N1-ICD expression in the E*µ*-TCL1 mouse model of CLL. **A** A schematic outline of the treatment schedule is shown. E*μ*-TCL1 cells were transplanted into C57BL/6 mice by intravenous (i.v.) injection. Twenty-eight days after transplantation (day 0), treatment started with DT-061 (5 mg/kg once daily for 28 days via oral gavage; *n* = 3) or vehicle (*n* = 3). Peripheral blood (PB) was harvested at the start of treatment (day 0), and at day +14 and day +28 from the start of the treatment. At day +28, mice were sacrificed, and spleen and bone marrow were collected. **B** The bar graphs with data points indicate the percentage of CD19^+^/CD5^+^ cells in PB from DT-061- and vehicle-treated mice, determined by flow cytometry. Data are presented as the mean ± SD of 3 mice per group. ^*^P < 0.05 according to unpaired Student’s *t*-test. **C**, **D** The bar graphs with data points indicate the number (left) and the percentage (middle) of CD19^+^/CD5^+^ cells in the spleen (**C**) and bone marrow (**D**) from DT-061- and vehicle-treated mice, determined by flow cytometry. Data are presented as the mean ± SD of 3 mice per group.^*^P < 0.05; ^***^P < 0.001 according to unpaired Student’s *t*-test. One representative dot plot of CD19/CD5 staining relative to each treatment is shown (right). **E**, **F** Left, bar graphs with data points indicate the percentage of viable Annexin V^−^ (An V^−^) cells in CD19^+^/CD5^+^ sorted from the spleen (**E**) and bone marrow (**F**) of DT-061- and vehicle-treated mice. Data are presented as the mean ± SD of 3 mice per group. ^*^P < 0.05 according to unpaired Student’s *t-*test. Middle, Western blot analysis of N1-ICD in CD19^+^/CD5^+^ cells sorted from the spleen (**E**) and bone marrow (**F**) of DT-061- and vehicle-treated mice performed using the anti-NOTCH1 Val1744 antibody. Right, bar graphs with data points of densitometric analysis of N1-ICD are shown. ^*^P < 0.05; ^**^P < 0.01 according to unpaired Student’s *t*-test.

We investigated whether DT-061 reduced the burden of CLL cells transplanted from E*µ*-TCL1 mice into C57BL/6 mice, and whether this effect was accompanied by changes in N1-ICD expression. Leukemic E*μ*-TCL1 cells were transplanted into C57BL/6 mice (*n* = 3), and DT-061 treatment started after 28 days (day 0), when mice had an average of 5–10% leukemic (CD19^+^/CD5^+^) cells in the peripheral blood (PB) (Fig. 7A, B). Mice received DT-061 or vehicle once daily for 28 days. PB was analyzed at days 0, 14 and 28 post-treatment, with tissues (spleen and bone marrow) harvested at day +28 for analysis (Fig. 7A). Flow cytometric analysis of PB samples showed that DT-061 induced a decrease in the percentage of CD19^+^/CD5^+^ cells compared with vehicle at day +28 (Fig. 7B). DT-061 administration was well tolerated, with no change in animal weight, hemoglobin levels and platelet count in treated mice (Supplementary Fig. S6). Analysis of the spleen and bone marrow showed that either the number or percentage of infiltrating leukemic CD19^+^/CD5^+^ cells were decreased in both organs from DT-061-treated mice compared with vehicle (Fig. 7C, D, respectively). Additionally, CD19^+^/CD5^+^ cells in both spleen (Fig. 7E, left) and bone marrow (Fig. 7F, left) of DT-061-treated mice exhibited a reduced viability compared with vehicle, as measured by Annexin-V single staining, suggesting that reduction in leukemic burden induced by DT-061 in these organs is due to decreased tumor cell survival. We then examined whether the observed antileukemic activity of DT-061 was accompanied by effects on N1-ICD levels. WB analysis of N1-ICD, performed in CD19^+^/CD5^+^ cells sorted from the spleen (Fig. 7E, middle and right) and bone marrow (Fig. 7F, middle and right) from DT-061-treated mice showed a reduction in N1-ICD levels compared with vehicle.

## Discussion

We provide molecular insights into NOTCH1 signaling regulation in CLL cells lacking *NOTCH1* mutation. By genetic and pharmacologic studies, we identified GSK3β as a critical component of the network regulating N1-ICD levels in CLL cells. In particular, we demonstrated that GSK3β silencing increases constitutive N1-ICD levels, whereas expression of an active GSK3β mutant reduces them, suggesting that GSK3β activity negatively regulates N1-ICD protein. Additionally, pharmacologic GSK3β inhibition by SB216763 promotes accumulation of N1-ICD and leukemic cell viability, suggesting that GSK3β is detrimental for CLL cell survival. Analysis of the mechanisms whereby SB216763 increases N1-ICD levels in CLL cells shows that this effect is due to increased N1-ICD stability, as a result of reduced proteasomal degradation. Specifically, co-immunoprecipitation and PLA assays demonstrated the presence of N1-ICD/GSK3β protein complexes in CLL cells, suggesting a possible interaction between the two proteins. Strikingly, SB216763 reduces GSK3β/N1-ICD interactions and the levels of ubiquitinated N1-ICD, suggesting that GSK3β downregulates NOTCH1 signaling by promoting N1-ICD proteasomal degradation.

Given that GSK3β inhibition increases NOTCH1 signaling and CLL cell survival, and NOTCH1 signaling is oncogenic in CLL [4], we suggest that GSK3β has a tumor suppressor role in CLL. Even other authors considered GSK3β as a tumor suppressor in CLL, as they showed that GSK3β inactivation by a deregulated SYK/PKCδ pathway stabilized the antiapoptotic Mcl-1 protein [59]. In line with these studies, it was demonstrated that antileukemic activity of the PKCβ inhibitor sotrastaurin (AEB071) in CLL, relies, besides PKCβ inhibition, on GSK3β activation associated with reduced expression of β-catenin and its targets c-myc, cyclin and CD44 [60]. Recently, it has been also shown that efficacy of the PI3Kδ/γ inhibitor duvelisib in combination with the Bcl-2 inhibitor venetoclax in Richter Syndrome (RS) patient-derived xenograft models, is due to GSK3β activation by PI3K inhibition which leads to c-myc and Mcl-1 degradation, making RS cells more sensitive to Bcl-2 inhibition [61]. In contrast, other authors proposed an oncogenic role for GSK3β in CLL, because its inhibition induces epigenetic silencing of NF-kB and increases CLL cell apoptosis [62]. Because of these controversies, the biological and clinical role of GSK3β in CLL should be better defined, in order to understand whether its targeting could be beneficial for the development of new strategies.

However, in malignancies where GSK3β plays a tumor suppressor role but is inactivated by oncogenic pathways through S9-phosphorylation [22–27], GSK3β-targeted strategies could rely on the suppression of the inhibitory pathways [32, 59–61]. One of them is PI3K/AKT which is aberrantly activated in CLL cells [47, 48], and contributes to CLL pathogenesis and progression [49]. In this context, a recent study demonstrated that genetic activation of AKT in the E*µ-*TCL1 mouse model of CLL drives CLL transformation to RS via overactivation of NOTCH1 signaling, triggered by increased expression of the NOTCH ligand DLL1 on CD4^+^ T cells [49]. Therefore, CLL progression to a more aggressive disease depends not only on *NOTCH1* mutations [63], but also on an aberrant NOTCH1 signaling activation fueled from the microenvironment [49]. Here, we demonstrate that AKT inhibition reduces NOTCH1 signaling and cell viability through activation of GSK3β in CLL cells lacking *NOTCH1* mutations, suggesting that GSK3β inactivation induced by AKT plays a role in promoting NOTCH1-associated survival. Considering that several microenvironment stimuli of CLL cells, including growth factors, cytokines, integrins and BCR, promote the activation of AKT and other kinases, that in turn inactivate GSK3β by S9 phosphorylation, GSK3β inactivation may represent a crucial event on which multiple extracellular signals converge for sustaining NOTCH1 signaling. In line with the hypothesis that N1-ICD is sustained by inactive GSK3β, we show a positive correlation between N1-ICD and pS9-GSK3β levels in CLL cells.

A positive regulator of GSK3β is PP2A which increases GSK3β activity by dephosphorylating it at S9 [28]. PP2A also inhibits several oncogenic pathways, representing an important tumor suppressor [28]. PP2A activity is impaired in many malignancies, thus promoting cancer progression and resistance to kinase-targeted therapies. PP2A activity is also impaired in CLL, mainly due to interaction with its physiological inhibitor SET, highly expressed in CLL cells [29, 30]. Therefore, even an impaired PP2A activity might sustain N1-ICD levels in CLL cells by contributing to GSK3β inactivation. In this regard, we show for the first time that DT-061, a highly specific PP2A activator, reduces N1-ICD levels through activation of GSK3β. Additionally, DT-061 induces apoptosis in CLL cells in vitro, as shown by the increased percentage of An V^+^ cells, increased PARP cleavage and reduced Mcl-1 levels. Strikingly, DT-061 also reduces leukemic burden in peripheral blood, spleen and bone marrow in the E*µ*-TCL1 mouse model of CLL, with a concomitant decrease in N1-ICD expression and viability of leukemic cells. These results highlight the relevance of PP2A-reactivating strategies in CLL by identifying in DT-061 a new drug with preclinical activity in CLL, that may have important implications, especially in those cases where kinase-targeted therapies fail to function. Indeed, an efficient inhibition of the oncogenic phosphorylation-dependent pathways could be achieved by combining kinase inhibitors with specific PP2A activators, as observed in other tumors [57, 58, 64]. Even the combination of DT-061 with venetoclax could hold promise, considering that one of the mechanisms of resistance to venetoclax in CLL is represented by the high expression of Mcl-1 [65], and that DT-061 reduces the levels of both Mcl-1 and NOTCH1 signaling, which has been shown to sustain Mcl-1 expression in CLL cells [7].

**Fig. 8:**
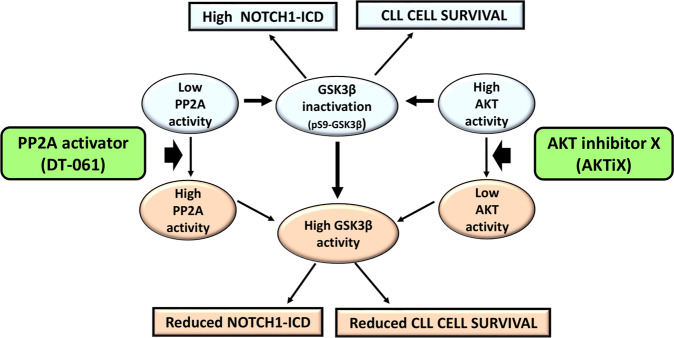
Schematic representation of the signaling network sustaining NOTCH1-ICD levels and cell survival in CLL, as a potential therapeutic target. Constitutive NOTCH1-ICD levels and CLL cell survival are sustained by GSK3β inactivation, due to an impaired PP2A activity and a high AKT activation, which both induce S9-GSK3β phosphorylation (pS9-GSK3β). The increase in PP2A activity induced by the highly specific activator DT-061 or the inhibition of AKT with the AKTiX inhibitor enhance GSK3β activity, leading to a decrease in NOTCH1-ICD levels with a concomitant reduction in CLL cell survival.

Overall, we show that GSK3β is a critical component of the network regulating N1-ICD stability and cell viability in CLL. Specifically, we reveal that increasing GSK3β activity, by manipulating its upstream regulators AKT and PP2A, reduces NOTCH1 signaling and CLL cell survival (Fig. 8).

The strategy to downregulate NOTCH1 activation by targeting its regulators could limit the detrimental effects caused by direct NOTCH1 inhibition and contribute to develop alternative therapeutic strategies not only for CLL but also for other NOTCH1-associated diseases.

## Materials and methods

### CLL patients and clinical laboratory characteristics

Forty-four CLL patients entered this study. Diagnoses of CLL were based on Stanford criteria defined by the National Cancer Institute-sponsored Working Group [66] and clinical staging was based on the Binet classification [67]. This study was approved by the local Ethics Committee, and all patients signed informed consent in accordance with Declaration of Helsinki. CD19^+^/CD5^+^ CLL cells were isolated from peripheral blood as previously reported [5–7]. All CLL samples contained >95% CD19^+^/CD5^+^ CLL cells, assessed by flow cytometry (EPICS-XL-MCL; Beckman Coulter, Fullerton, CA)*. IGHV* mutations, CD38 and ZAP70 expression, cytogenetic abnormalities, and *NOTCH1* exon 34 mutational status were analyzed as reported [5, 13, 21, 68]. Supplementary Table S1 gives the clinical and biological characteristics of CLL patients. All patients were wild-type for *NOTCH1*.

### In vitro CLL cell treatments

In pharmacologic studies, CLL cells (2 × 10^6^/ml) were cultured for different times in complete medium, consisting of RPMI 1640 supplemented with 10% heat-inactivated fetal bovine serum (Gibco), 2mM L-glutamine, 100U/ml penicillin and 100 μg/ml streptomycin (all from Invitrogen), with the following agents: the GSK3β inhibitor SB216763, the AKT inhibitor X (AKTiX) (both from Calbiochem, La Jolla, CA); the proteasome inhibitor MG132 and the translation inhibitor cycloheximide (both from Sigma-Aldrich, St. Louis, MO); the PP2A activator DT-061 and the PI3Kδ inhibitor CAL-101 (both from Selleck Chemicals, Houston, TX). In BCR stimulation experiments, CLL cells from *IGHV*-unmutated patients were stimulated for 15 min with 10 μg/ml of immobilized AffiniPure F(ab’)2 Fragment Goat Anti-Human IgG+IgM (H + L) (Jackson ImmunoResearch Laboratories, West Grove, PA) or isotype control.

### Plasmids and gene transfection

The plasmids (pcDNA3) containing the hemagglutinin (HA)-tagged constitutively active (S9A) GSK3β, originally created by Dr. Jim Woodgett’s laboratory [43], were obtained from Addgene (Cambridge, MA; plasmid 14754). The pcDNA3.1 empty vector was used as control. Transient transfection of CLL cells was performed using the *Trans*IT-X2 Dynamic Delivery System (Mirus Bio, Madison, WI) according to manufacturer’s instructions. Briefly, CLL cells were cultured at 2 × 10^6^/ml for well in a 12-well plate in the presence of 1 μg/well of plasmid diluted in OptiMEM I Reduced-Serum Medium with the appropriated volume of *Trans*IT-X2 reagent (4:1 transfection reagent to DNA ratio) in a 103 μl volume reaction per well added dropwise. After 48 h CLL cells were harvested and analyzed for the expression of N1-ICD and GSK3β protein.

### siRNA transfection

To downregulate GSK3β expression, CLL cells were transfected using the Amaxa nucleofection technology and the ON-TARGETplus SMARTpool small interfering RNA (siRNA) to GSK3β (siGSK3β) or ON-TARGETplus siCONTROL nontargeting pool (siCtrl) as negative control (Dharmacon RNA Technologies). CLL cells (12 × 10^6^) were resuspended in 100 µl Cell Line Solution Kit V (Lonza Group Ltd) with 0.25 μM of siGSK3β or siCtrl, transferred to the cuvettes and transfected with the Amaxa Nucleofector II/2b device (program U-013). Cells were immediately transferred into 12-well plates in complete medium, and after 48 h were examined for the expression of N1-ICD, and GSK3β protein to verify the efficiency of silencing.

### Western blot and co-immunoprecipitation assay

Whole-cell lysate extraction and Western blot were performed as previously reported [5–7], using the primary antibodies listed in Supplementary Table S2. Primary antibodies were detected using horseradish peroxidase-linked secondary antibodies (Jackson ImmunoResearch Laboratories) together with the ECL system (Sigma-Aldrich) or SuperSignal™ West Pico PLUS Chemiluminescent Substrate (Thermo Fisher Scientific). Densitometric analysis was performed using Quantity One or Image Lab software (Bio-Rad). Scans of uncropped Western blots are shown in Supplementary Information.

For co-immunoprecipitation assay, whole-cell lysates (4 mg) were incubated with 20 µl Protein A/G Plus Agarose beads (Santa Cruz Biotechnology, Santa Cruz) with gentle rocking at 4 °C for 1 h for pre-clearing. The beads were pelleted, and the lysates were recovered and incubated at 4 °C overnight with 6 µg of the rabbit monoclonal anti-NOTCH1 antibody (Val1744; clone D3B8, Cell Signaling Technology) or normal rabbit IgG antibody as negative control (Cell Signaling Technology). Beads (50 µl) were then added to pull-down the immune complexes and incubation was continued for 1.5 h. After centrifugation, the beads carrying immune complexes were washed six times in wash buffer (10 mM Tris-HCl pH 7.5, containing 150 mM NaCl, 5 mM EGTA, 0.1% IGEPAL CA-630 and complete protease and phosphatase inhibitor cocktail), and then pelleted and resuspended in 50 µL 2X Laemmli sample buffer containing 2-mercaptoethanol and boiled for 5 min at 95 °C for elution of the immune complexes. Immune complexes were analyzed by Western blot with the anti-NOTCH1 antibody (Val1744) as control, and the anti-GSK3β antibody (clone 3D10) to detect GSK3β/N1-ICD interactions.

### Flow cytometric analysis of N1-ICD

CLL cells were fixed and permeabilized with the Fix & Perm Kit, and then incubated with mouse anti-NOTCH1 (mN1A)-PE antibody or mouse IgG1 kappa isotype control (all from Thermo Fisher Scientific). Flow cytometry data were analyzed by FlowJo software.

### Quantitative Real-time PCR

RNA was extracted using RNeasy Plus Kit (Qiagen, Hilden, Germany), and cDNA was obtained using Prime Script RT Master Mix (Takara Bio, Dalian, China). Real-time quantitative PCR was performed with PCR Master Mix Power SYBR Green (Applied Biosystems, Warrington, UK), using the 7900HT Fast Real-Time PCR System (Applied Biosystems). The sequences of primers for NOTCH1, HES1, DELTEX and GAPDH (all from Thermo Fisher Scientific) analysis are shown in Supplementary Table S3. The expression of each target gene was normalized to GAPDH, and relative fold change was calculated using the 2-ΔΔCt method.

### Analysis of cell viability

Cell viability was evaluated by MTS assay as previously described [6] and by flow cytometry after Annexin V/propidium iodide (An V/PI) double staining, performed with a commercial kit (Immunotech, Beckman Coulter, Marseille, France), according to manufacturer’s instructions. Results were analyzed by FlowJo software. Data of MTS assays, performed in CLL cells treated with different concentrations of AKTiX and SB216763 as single agents or in combinations, were analyzed by the SynergyFinder web application 3.0 [51], in order to evaluate the antagonistic effect of SB216763 on AKTiX. The score was calculated by using the ZIP Synergy model. A score lower than −10 indicates an antagonistic effect (green area), between −10 and +10 indicates an additive effect (white area), and higher than +10 indicates a synergistic effect (red area).

### Immunofluorescence and confocal microscopy analysis

Immunofluorescence analysis was performed to detect subcellular localization of GSK3β. Cells (2 × 10^5^) were seeded on poly-l-lysine-coated glass slides (Thermo Fisher Scientific) and fixed with a 4% paraformaldehyde solution in phosphate buffer saline (PBS) for 10 min at room temperature. Cells were then permeabilized with 0.1% Triton X-100 in PBS for 5 min at room temperature. After three washes in PBS with 0.01% Triton X-100, cells were blocked with blocking buffer (1% bovine serum albumin [BSA] in PBS) for 30 min, before overnight incubation at 4 °C with the mouse anti-GSK3β antibody (clone 3D10) diluted 1:100 in blocking buffer. After three washes in PBS with 0.01% Triton X-100, cells were incubated with goat anti-mouse Alexa-Fluor 568 for 40 min in the dark. Nuclei were stained with 4,6-DiAmidino-2- Phenyl Indole (DAPI) in ProLong Gold antifade mounting reagent (Thermo Fisher Scientific). Images were acquired with a laser scanning confocal microscope LSM 800 with Airyscan (Zeiss) using a 63x oil immersion and 1.4 NA objective. Scale bar, 10 μm.

### Proximity Ligation Assay and confocal microscopy analysis

The Proximity Ligation Assay (PLA) was applied to examine the interactions between N1-ICD and GSK3β and between N1-ICD and ubiquitin in CLL cells. PLA was performed on fixed CLL cells with Duolink® PLA technology probes and reagents (Sigma-Aldrich), following the manufacturers protocol. Briefly, CLL cells were permeabilized in 0.1% Triton X-100 in PBS, blocked in Duolink® Blocking Solution and incubated overnight at 4 °C with rabbit anti-NOTCH1 (Val1744; clone D3B8) and mouse anti-GSK3β (clone 3D10) antibodies, or with rabbit anti-NOTCH1 (Val1744; clone D3B8) and mouse anti-ubiquitin (clone FK2) antibodies diluted in Duolink® Antibody Diluent solution. Incubation with Duolink® MINUS and PLUS probes conjugated to secondary antibody, ligation, and amplification steps for PLA were performed as suggested by the manufacturer using 40 μl volume reaction. Following amplification, slides were washed for 10 min in Buffer A and B, and then mounted with Duolink® in situ mounting medium containing DAPI. Negative controls were obtained by omitting primary antibodies. Fluorescent images were obtained using a confocal microscope LSM 800 (Zeiss) with Airyscan using a 63x oil immersion and 1.4 NA objective. Scale bar, 10 μm.

### E*µ*-TCL1 adoptive transfer model

C57BL/6 mice (12-weeks old), 24 h after a sub-lethal irradiation (4.5 Gy), received frozen splenocytes (2 × 10^7^ per mouse) from E*µ*-TCL1 donors by intravenous injection. Treatment started (day 0) when mice exhibited 5–10% CD19^+^/CD5^+^ leukemic cells in peripheral blood (28 days after transplant). Mice received 5 mg/kg of DT-061 (Selleck Chemicals) once daily for 28 days by oral gavage. Control group received 10% DMSO, 40% PEG-300, 5% Tween-80 and 45% ddH_2_O, as a vehicle. Peripheral blood was recovered at day 0, day +14 and day +28 from anesthetized mice, and then analyzed with a DxH520 hematology analyzer (Beckman Coulter). Spleen and bone marrow were recovered at day +28 from euthanized mice, and cell suspensions were analyzed by flow cytometry for CLL murine markers, CD19 and CD5 (Miltenyi Biotec Inc.). Cell-sorting of CD19^+^/CD5^+^ cells was performed using BD FACS-Aria III (BD Biosciences). Cell viability was assessed after Annexin V staining (Immunotech). The Institutional Animal Care and Use Committee approved all the procedures. Mice were treated following the European guidelines and with the approval of the Italian Ministry of Health (authorization #971/2020-PR).

### Statistical analyses

Statistical analyses were performed with GraphPad v8 (GraphPad Software Inc.). Statistical differences between mean values were evaluated using the non‐parametric Wilcoxon paired test. In animal studies and confocal microscopy analyses, an unpaired Student’s *t*-test was used. Results were considered statistically significant with *P*-value < 0.05.

## Supplementary information

Uncropped blots

Supplementary Information

Change of Authorship Request

Reproducibility Checklist

## Data Availability

All data and information concerning this study will be made available from the corresponding authors upon request.
