# Supplementary material for: GSK3β is a critical, druggable component of the network regulating the active NOTCH1 protein and cell viability in CLL
Source: Cell Death Dis. 2022 Sep 1;13(9):755. doi: 10.1038/s41419-022-05178-w (PMC9436923; doi:10.1038/s41419-022-05178-w)

Uncropped blots shown in Figure 1A

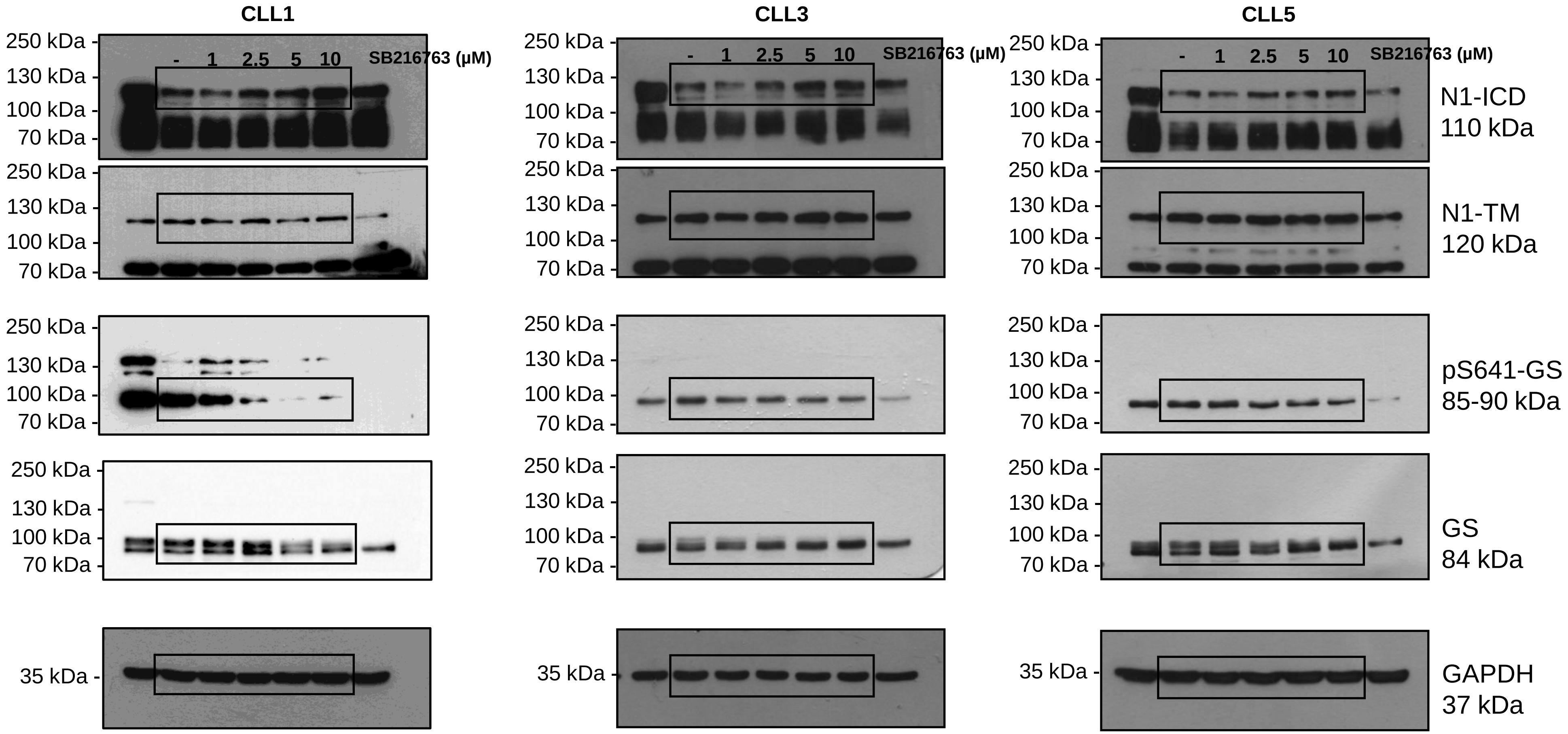

Uncropped blots shown in Figure 1C

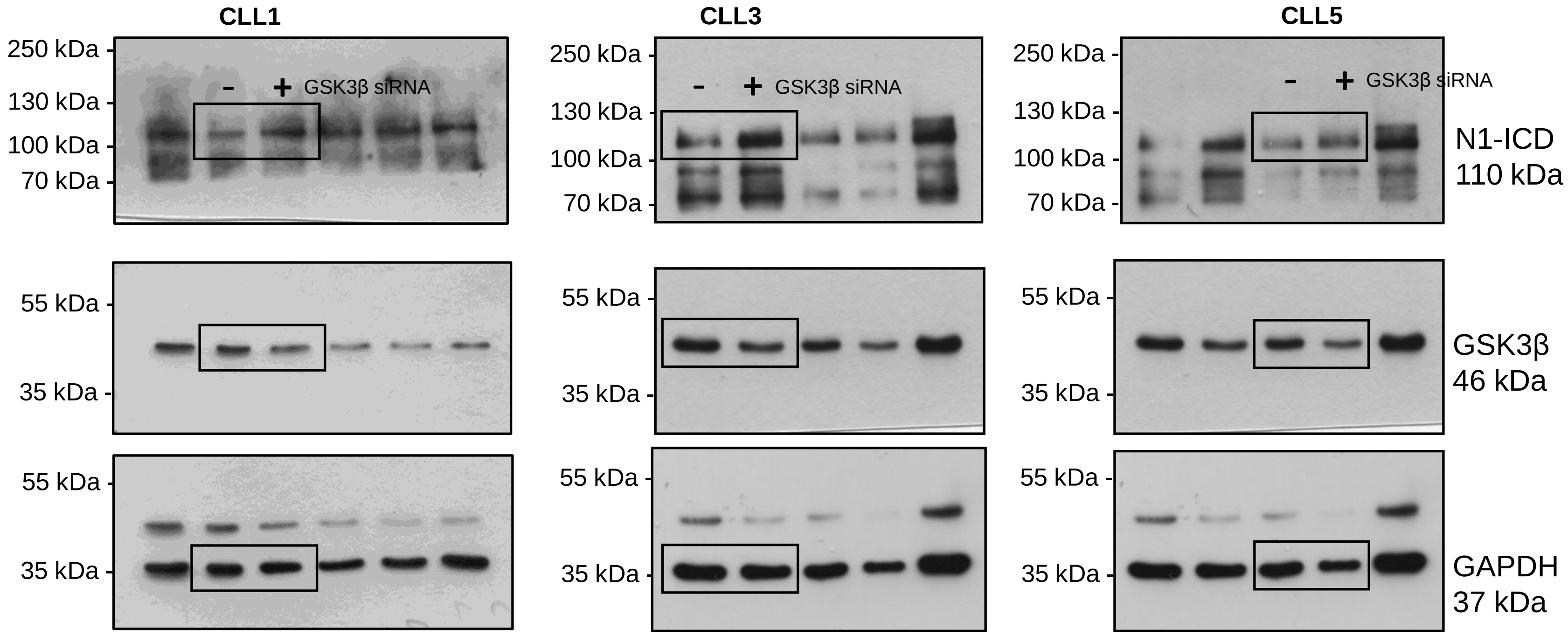

Uncropped blots showed in Figure 1D

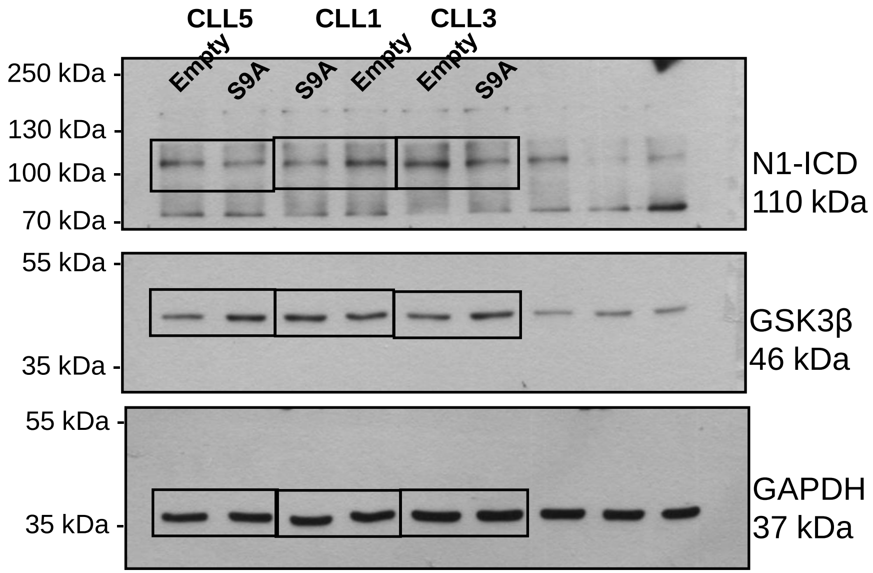

Uncropped blots shown in Figure 2B

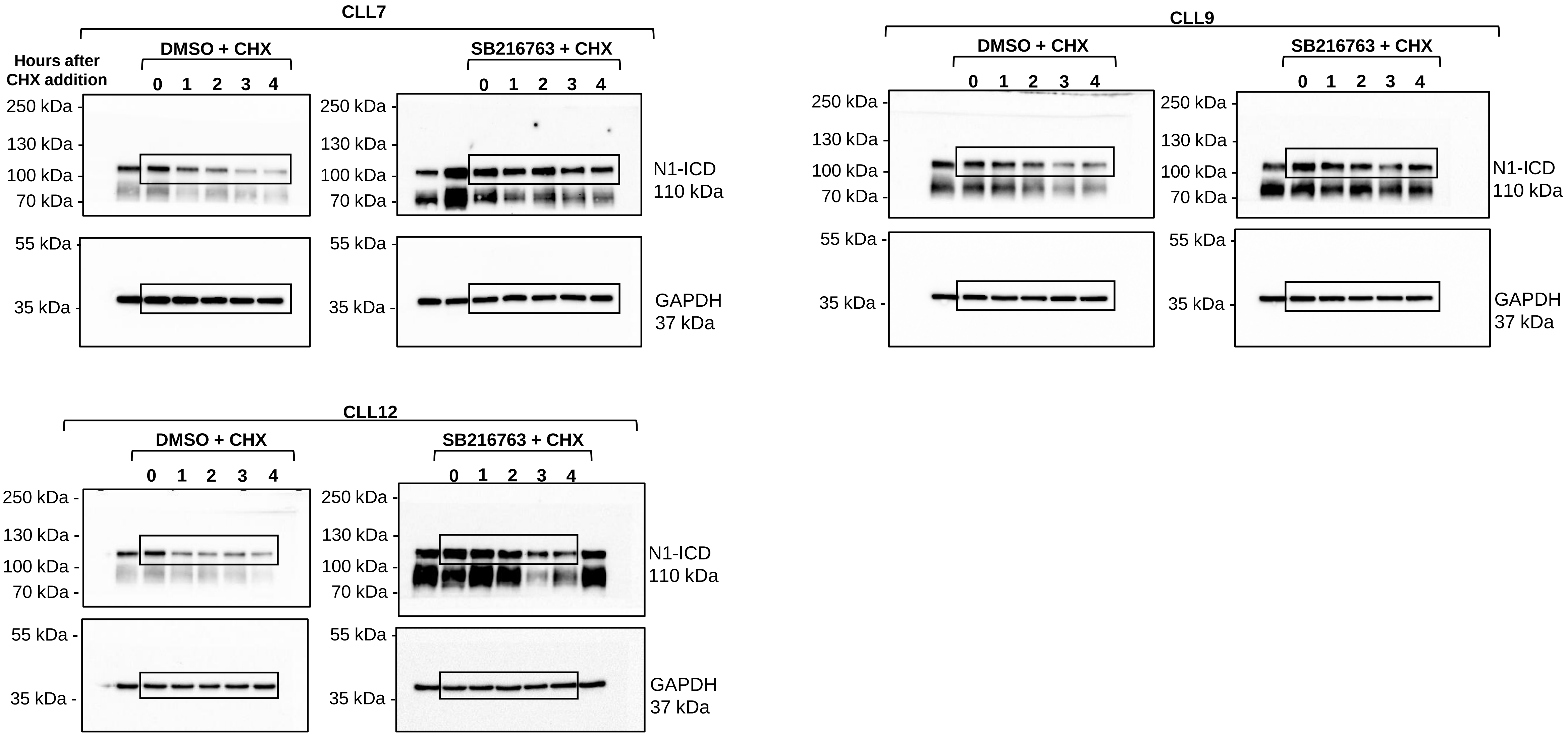

Uncropped blots shown in Figure 3A

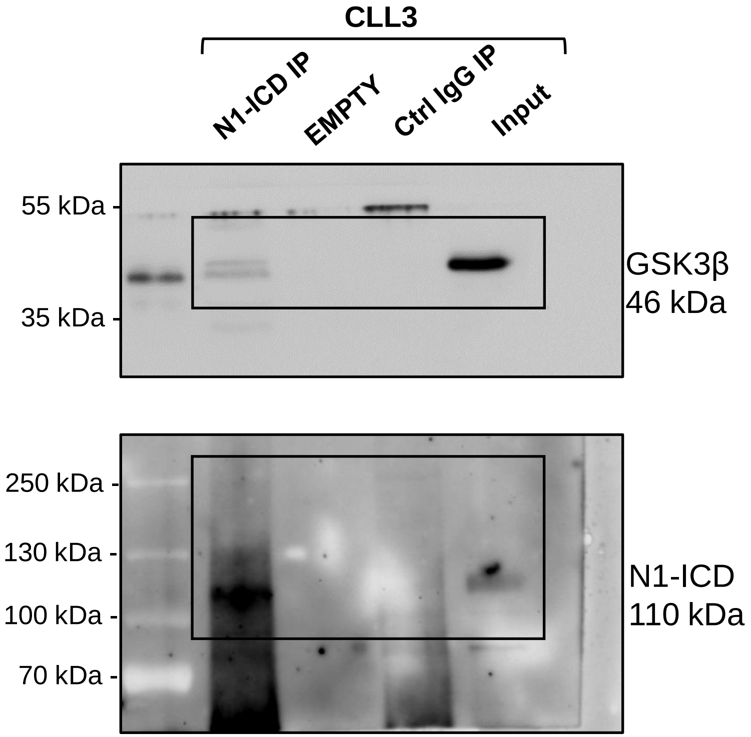

Uncropped blots shown in Figure 4

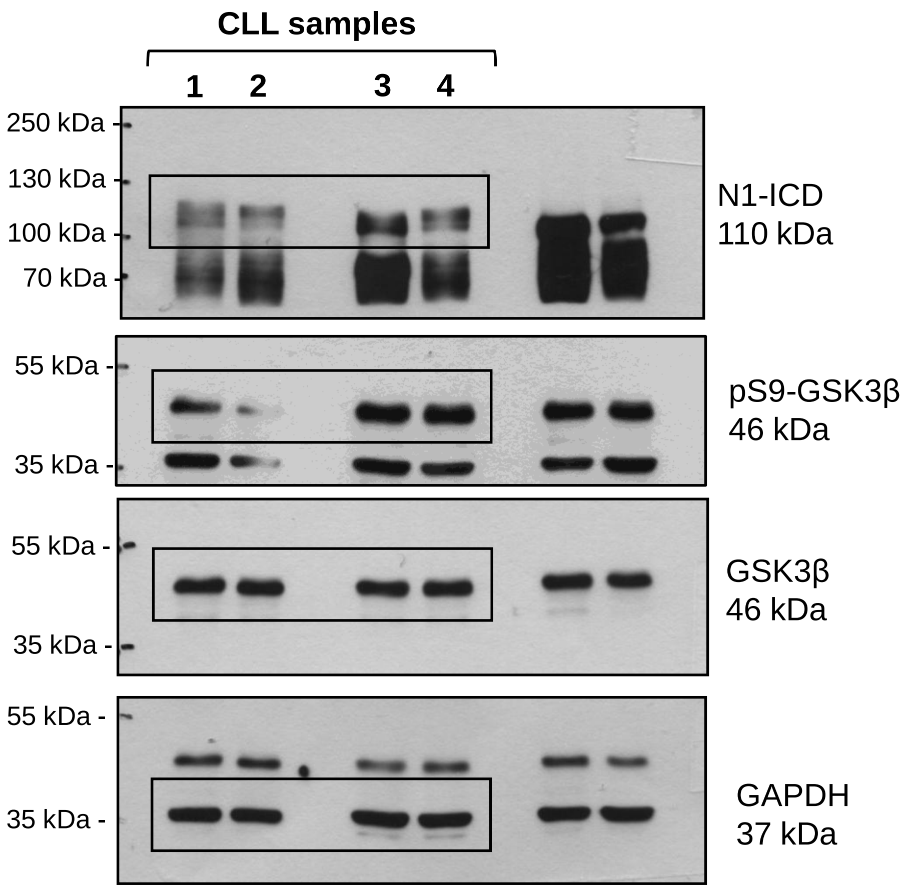

Uncropped blots shown in Figure 5A

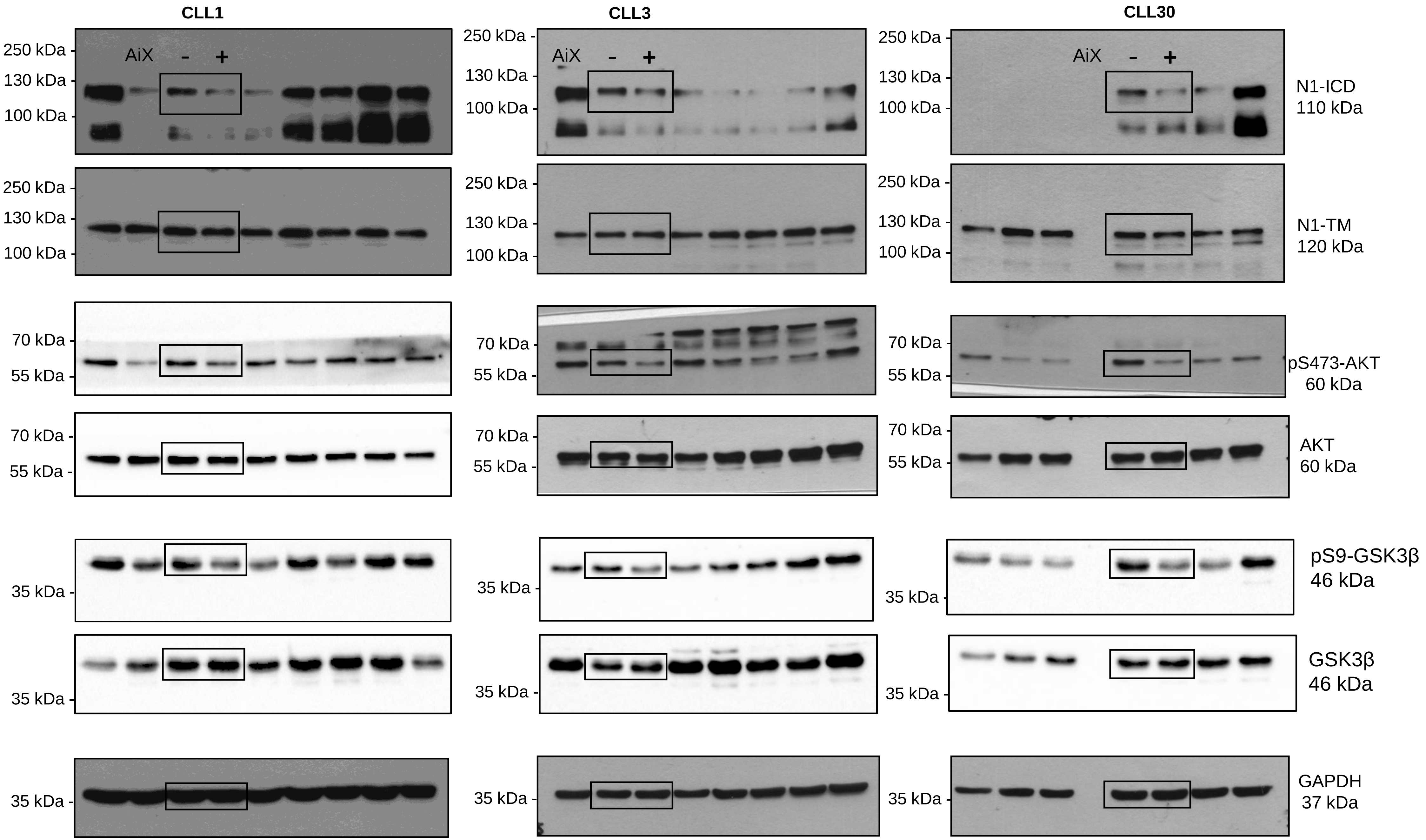

Uncropped blots shown in Figure 5B

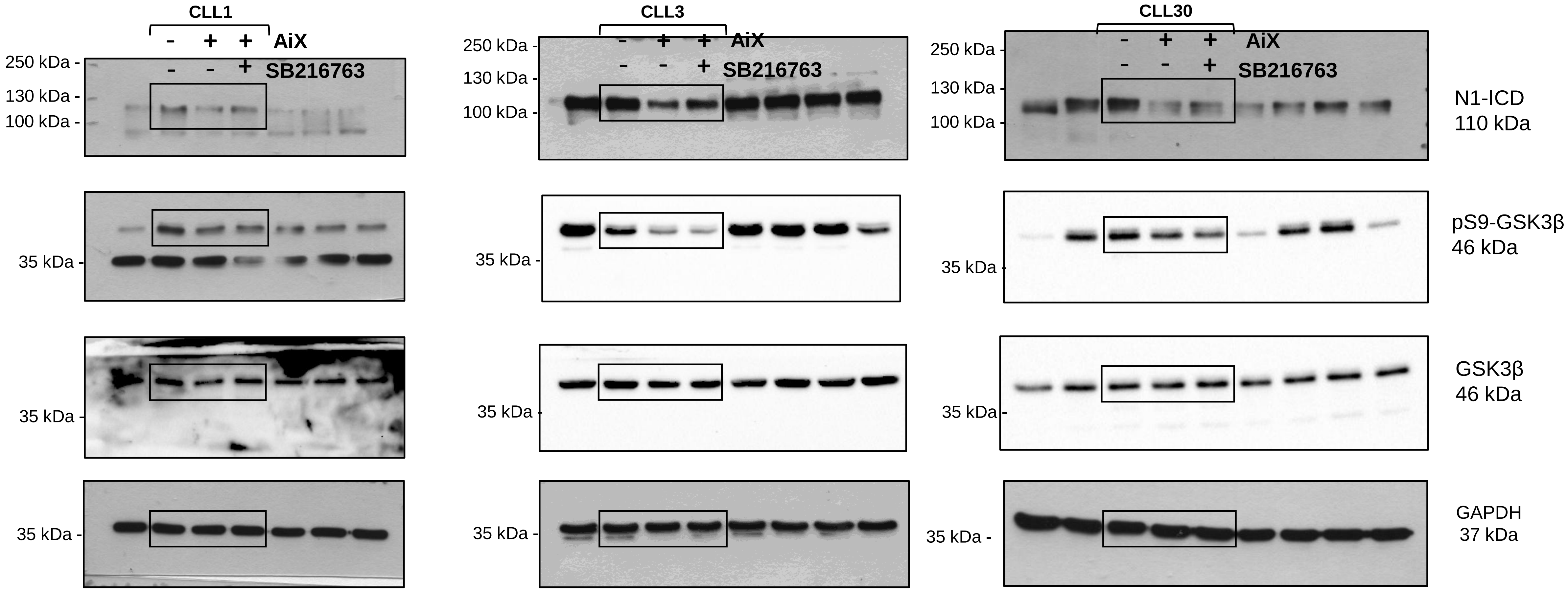

Uncropped blots shown in Figure 5C

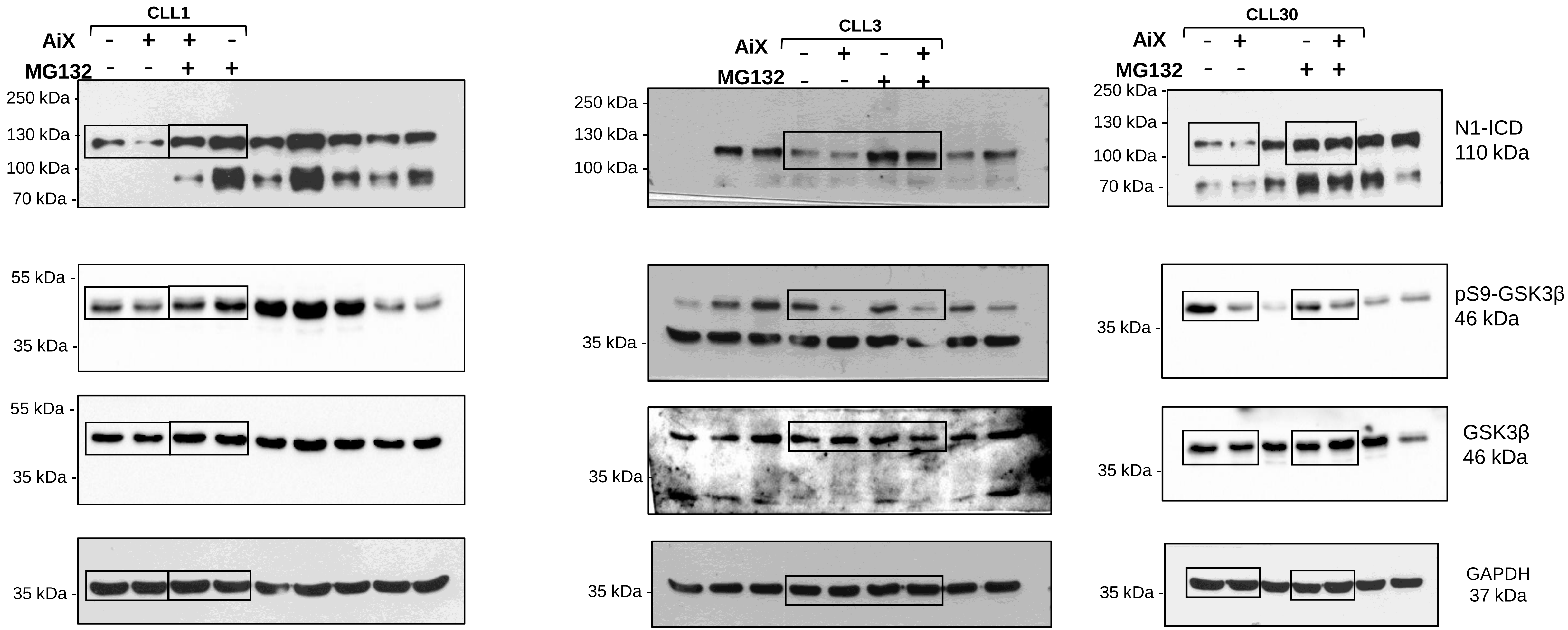

Repositioned gel lanes

Repositioned gel lanes

Uncropped blots shown in Figure 6B

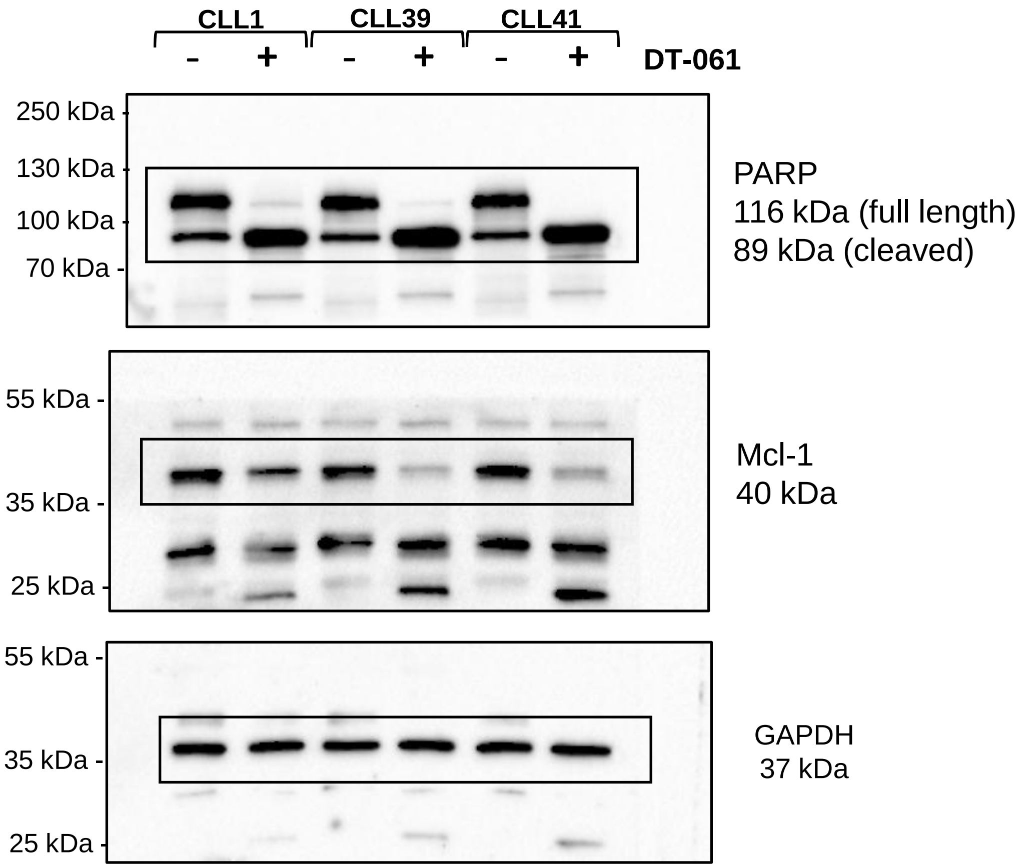

Uncropped blots shown in Figure 6D

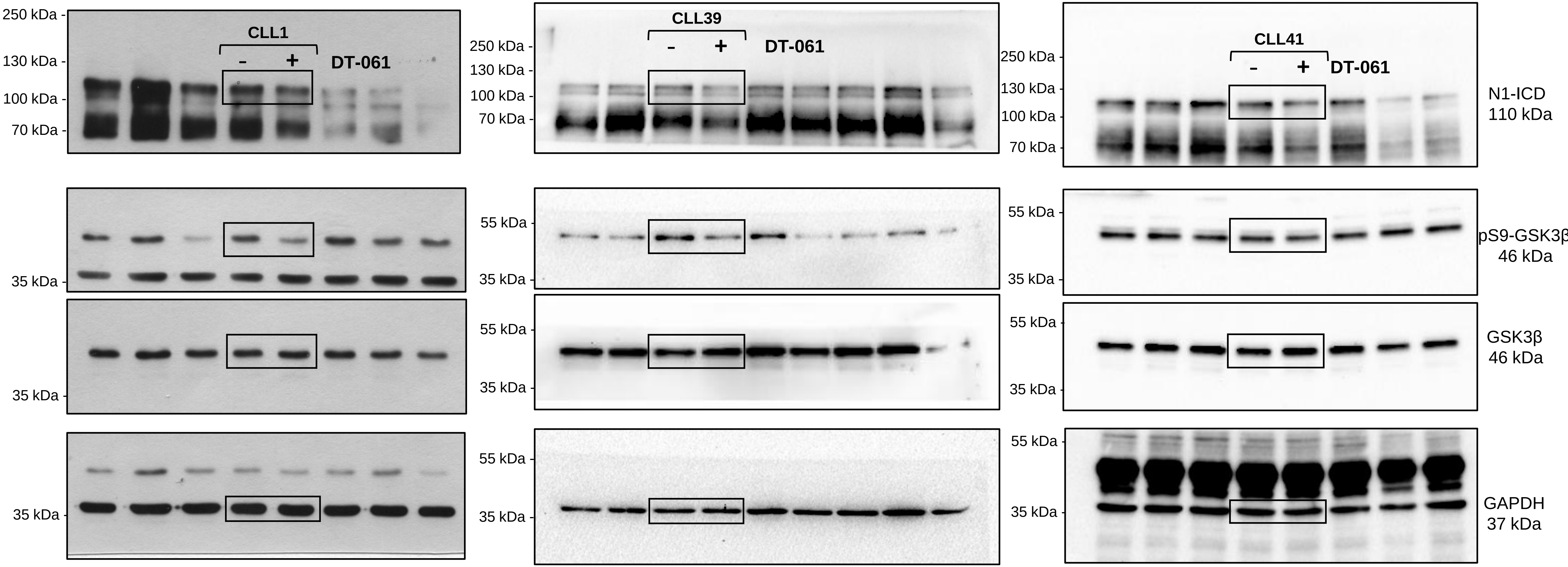

Uncropped blots shown in Figure 6E

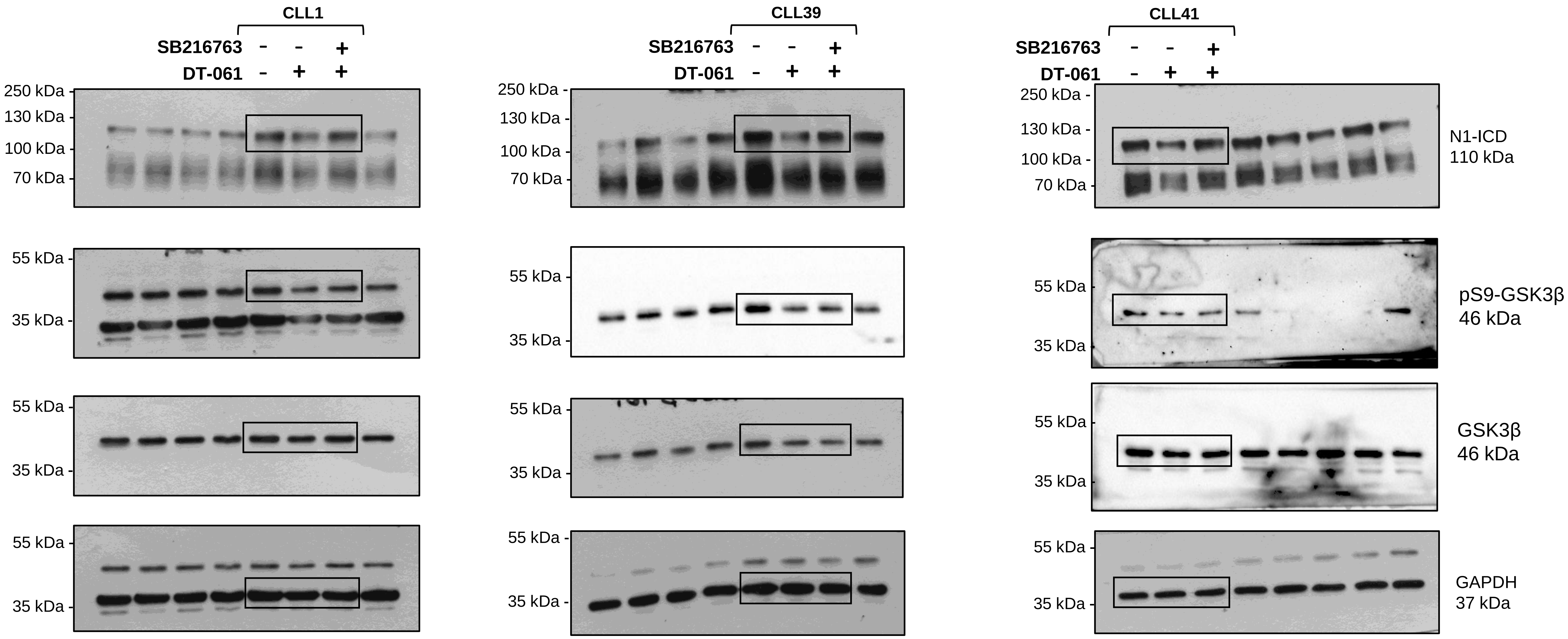

Uncropped blots shown in Figure 7E

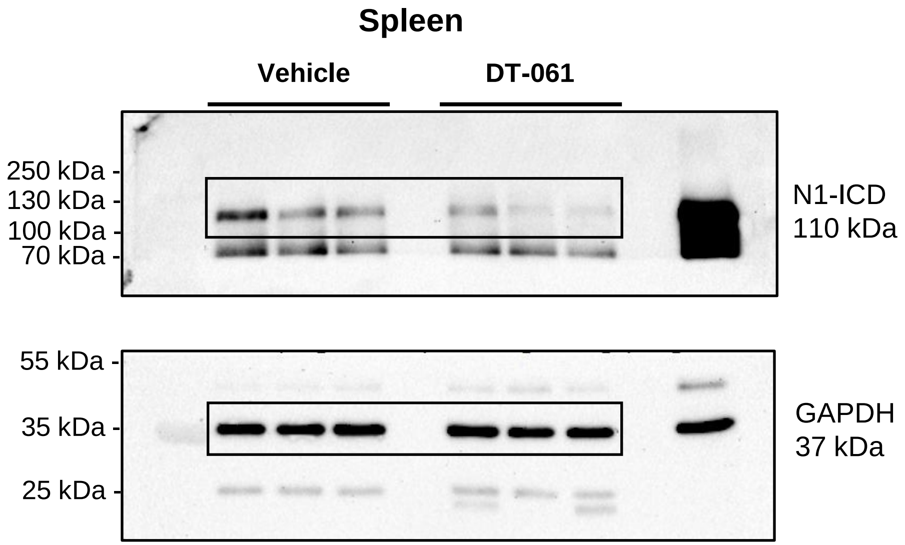

Uncropped blots shown in Figure 7F

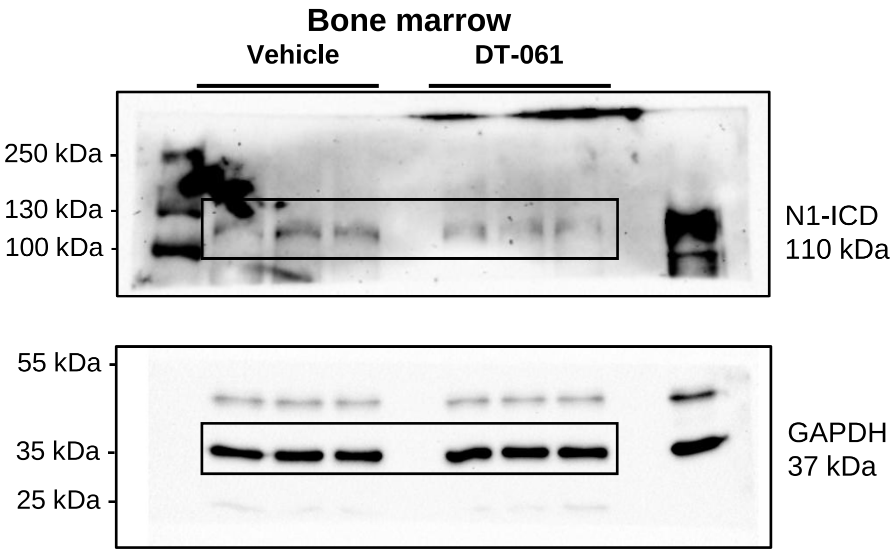

Supplement: Supplementary file 1 — Uncropped blots [file 41419_2022_5178_MOESM1_ESM.pdf]
