# Supplementary material for: GSK3β is a critical, druggable component of the network regulating the active NOTCH1 protein and cell viability in CLL
Source: Cell Death Dis. 2022 Sep 1;13(9):755. doi: 10.1038/s41419-022-05178-w (PMC9436923; doi:10.1038/s41419-022-05178-w)
Supplement: Supplementary file 2 — Supplementary Information [file 41419_2022_5178_MOESM2_ESM.pdf]

Supplementary Information

GSK3β is a critical, druggable component of the network regulating the active NOTCH1 protein and cell viability in CLL

Filomena De Falco, Chiara Rompietti, Daniele Sorcini, Angela Esposito, Annarita Scialdone, Stefano Baldoni, Beatrice Del Papa, Francesco Maria Adamo, Estevão Carlos Silva Barcelos, Erica Dorillo, Arianna Stella, Mauro Di Ianni, Isabella Screpanti, Paolo Sportoletti, and Emanuela Rosati

Contents:

- Supplementary Tables:

Supplementary Table S1: Characteristics of CLL patients

Supplementary Table S2: List of antibodies

Supplementary Table S3: Sequences of primers used for RT-PCR

- Supplementary Figures:

Supplementary Figure S1. Pharmacologic GSK3β inhibition enhances N1-ICD levels in CLL cells

Supplementary Figure S2. Pharmacologic GSK3β enhances N1-ICD stability in CLL cells

Supplementary Figure S3. N1-ICD is degraded by proteasome in CLL cells

Supplementary Figure S4. The PI3Kδ inhibitor CAL-101 reduces N1-ICD levels in CLL cells

Supplementary Figure S5. Effect of AKT inhibition on BCR-induced N1-ICD levels in CLL cells

Supplementary Figure S6. Effect of DT-061 treatment on animal weight, hemoglobin levels and platelet count in the Eμ-TCL1 mouse model of CLL

Supplementary Table S1. Characteristics of CLL patients

**Patients**	**Binet stage**	***IGHV* status[a]**	**ZAP70 expression[b]**	**CD38 expression[c]**	**Cytogenetic alterations[d]**
**CLL1**	A	Mut	Neg	Neg	del 13
**CLL2**	A	Mut	Neg	Pos	tris 12
**CLL3**	A	Unm	Neg	Neg	Normal
**CLL4**	A	Mut	Neg	Neg	del 13
**CLL5**	C	Mut	Pos	Pos	Normal
**CLL6**	C	Mut	Pos	Neg	del 13
**CLL7**	C	Unm	Pos	Neg	del 11 del13
**CLL8**	B	Unm	Pos	Neg	del 17
**CLL9**	B	Unm	Pos	Neg	del 13 del 14
**CLL10**	A	Mut	Pos	Neg	Normal
**CLL11**	A	Mut	Neg	Neg	Normal
**CLL12**	A	Mut	Neg	Neg	Normal
**CLL13**	A	Unm	Pos	Neg	del 13 del 14
**CLL14**	A	Mut	Pos	Neg	ND
**CLL15**	A	Mut	Neg	Neg	del 13
**CLL16**	B	Unm	Pos	Neg	tris 12 del13
**CLL17**	A	Unm	Neg	Neg	Normal
**CLL18**	B	Mut	Neg	Neg	Normal
**CLL19**	B	Mut	Neg	Neg	del 13 del 14
**CLL20**	A	Unm	Neg	Neg	ND
**CLL21**	B	Mut	Pos	Neg	Normal
**CLL22**	A	Unm	ND	Pos	del 14
**CLL23**	B	Unm	Pos	Pos	del 11 del13
**CLL24**	A	Mut	Neg	Neg	ND
**CLL25**	A	Unm	Pos	Neg	Normal
**CLL26**	B	Unm	Neg	Neg	del 11 del13
**CLL27**	A	Mut	Neg	Neg	del 13
**CLL28**	A	Mut	Neg	Neg	del 13
**CLL29**	B	Unm	Neg	Neg	del 13 del 17
**CLL30**	A	Mut	Neg	Neg	Normal
**CLL31**	B	Mut	Pos	Neg	del 13
**CLL32**	A	Mut	Pos	Neg	del 13 del 14
**CLL33**	A	Mut	Neg	Pos	del 13
**CLL34**	C	Mut	Neg	Neg	Normal
**CLL35**	B	Mut	Pos	Pos	del 13 del 14
**CLL36**	B	Unm	Neg	Neg	del 14
**CLL37**	B	Unm	Neg	Neg	del 13
**CLL38**	A	Unm	Pos	Neg	del13
**CLL39**	A	Unm	Pos	Neg	del 13
**CLL40**	A	Mut	Neg	Neg	del 13
**CLL41**	B	Mut	Neg	Neg	del 13

**CLL42**	A	Unm	Neg	Neg	del 13
**CLL43**	A	Mut	Neg	Neg	del 13
**CLL44**	A	Unm	Neg	Pos	tris 12

Abbreviations: Mut, mutated; Neg, negative; ND, not determined; Pos, positive; Unm, unmutated.

[a] Mutated was defined as having a frequency of mutations >2% from germline *VH*.
[b] Positivity refers to detection of >20% ZAP70$^+$/CD19$^+$.
[c] Positivity refers to detection of >20% CD38$^+$/CD19$^+$.
[d] Assessed by FISH.

Supplementary Table S2. List of antibodies

Target protein	Clone	Species	Supplier	Catalog number	Application
NOTCH1-ICD (Val1744)	D3B8	Rabbit	CST	#4147	WB<br>co-IP<br>PLA
NOTCH1-TM	D1E11	Rabbit	CST	#3608	WB
NOTCH1-ICD	mN1A	Mouse	ThermoFisher Scientific	#12-5785-82	Flow cytometry
Phospho-GS (Ser641)	D4H1B	Rabbit	CST	#47043	WB
GS	15B1	Rabbit	CST	#3886	WB
Phospho-GSK3β (Ser9)	5B3	Rabbit	CST	#9323	WB
GSK3β	3D10	Mouse	CST	#9832	WB<br>co-IP<br>IF<br>PLA
Ubiquitin	FK2	Mouse	Enzo Life Sciences	BML-PW8810	PLA
Phospho-AKT (Ser473)	D9E	Rabbit	CST	#4060	WB
AKT	Polyclonal	Rabbit	CST	#9272	WB

Mcl-1	D35A5	Rabbit	CST	#5453	WB
PARP	Polyclonal	Rabbit	CST	#9542	WB
GAPDH	GAPDH-71.1	Mouse	Sigma	G8795	WB

Abbreviations: CST, Cell Signaling Technology; WB, western blot; co-IP, co-immunoprecipitation; IF, immunofluorescence; PLA, proximity ligation assay.

Supplementary Table S3. Sequences of primers used for RT-PCR

Gene name	Forward primer (5' to 3')	Reverse primer (5' to 3')
**NOTCH1**	GAGGCGTGGCAGACTATGC	CTTGTACTCCGTCAGCGTGA
**HES1**	AAGAAAGATAGCTCGCGGCAT	CCAGCACACTTGGGTCTGT
**DTX1**	CAGCCGCCTGGGAAGATGGAG	TGGATGCCTGTGGGGATGTCA
**GAPDH**	ATGGGGAAGGTGAAGGTCG	GGGGTCATTGATGGCAACAATA

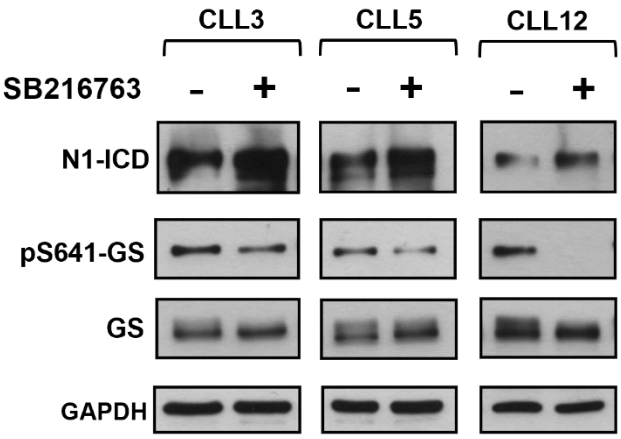

Supplementary Figure S1. Pharmacologic GSK3β inhibition enhances N1-ICD levels in CLL cells. CLL cells were cultured for 1.5 h with 5μM SB216763 or DMSO as control (n=3). Western blot analysis of NOTCH1 was performed using the anti-NOTCH1 (Val1744) antibody able to recognize N1-ICD. GSK3β activity inhibition by SB216763 was assessed by analyzing the glycogen synthase phosphorylation at Serine 641 (pS641-GS). Protein loading was assessed using an anti-GAPDH antibody.

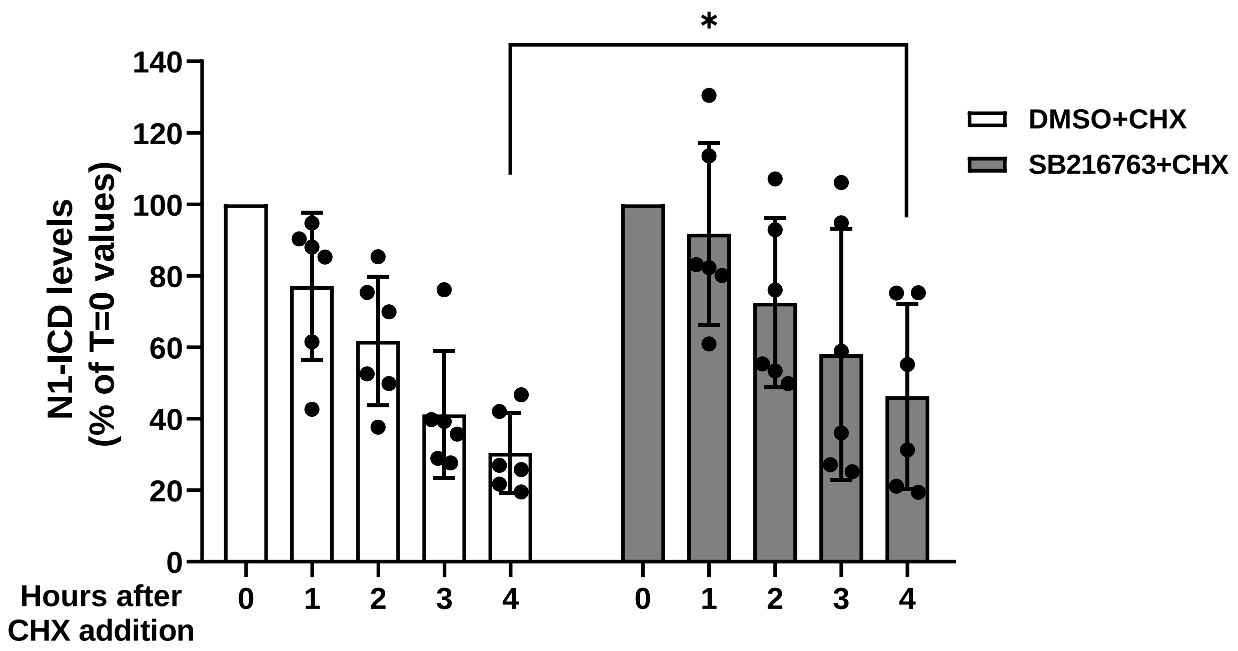

Supplementary Figure S2. Pharmacologic GSK3β inhibition enhances N1-ICD stability in CLL cells. After pretreatment with 5μM SB216763 or DMSO for 1.5 h, CLL cells were treated (T=0) with 50μg/ml CHX and harvested at the indicated times (n=6) for Western blot analysis of N1-ICD and GAPDH, as loading control. N1-ICD bands were quantified by densitometric analysis, normalized to GAPDH and represented as percentage of T=0 value set to 100%. Bar graphs with data points of densitometric analysis of N1-ICD are shown. Data are presented as the mean ± SD of 6 CLL samples. *$P < 0.05$ according to Wilcoxon paired test.

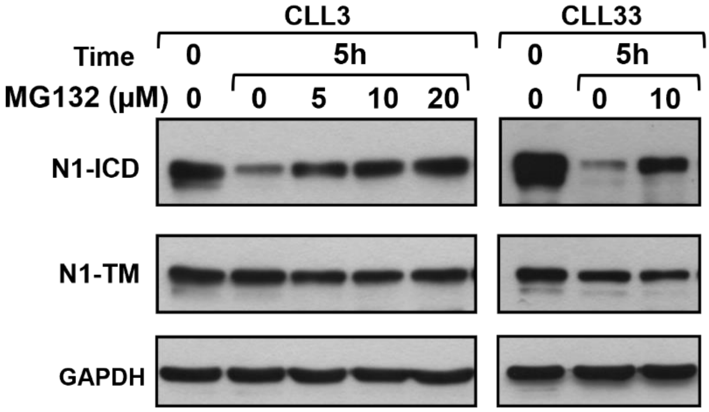

Supplementary Figure S3. N1-ICD is degraded by proteasome in CLL cells. CLL cells were cultured with the indicated concentrations of MG132 for 5 h or DMSO as control (n=3). Western blot analysis of NOTCH1 was performed using the anti-NOTCH1 (Val1744) and the anti-NOTCH1 (D1E11) antibodies, able to detect N1-ICD and N1-TM, respectively. Protein loading was assessed using an anti-GAPDH antibody. Two representative CLL samples are shown.

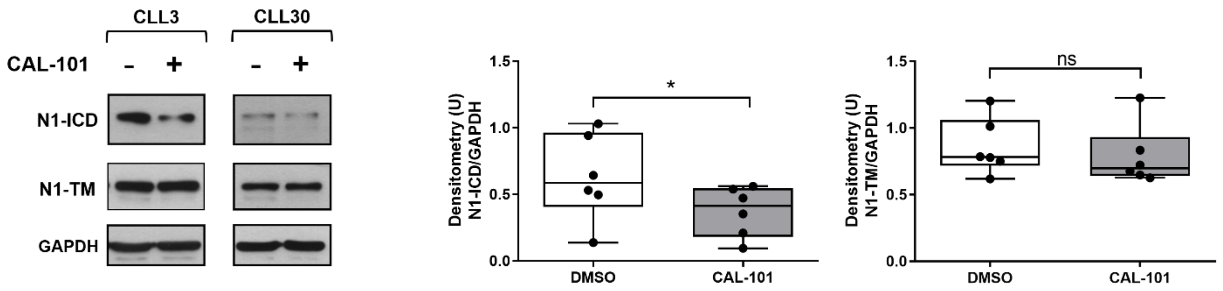

Supplementary Figure S4. The PI3Kδ inhibitor CAL-101 reduces N1-ICD levels in CLL cells. CLL cells were cultured for 6 h with 5μM CAL-101 or DMSO (n=6). Western blot analysis of NOTCH1 was performed using the anti-NOTCH1 (Val1744) and the anti-NOTCH1 (D1E11) antibodies, able to recognize N1-ICD and N1-TM, respectively. Protein loading was assessed by using an anti-GAPDH antibody. Left, two representative CLL samples are shown. Right, box and whisker plots with data points of densitometry analysis of N1-ICD and N1-TM are shown. Densitometry units (U) were calculated relative to GAPDH. *P < 0.05; ns = not significant according to Wilcoxon paired test.

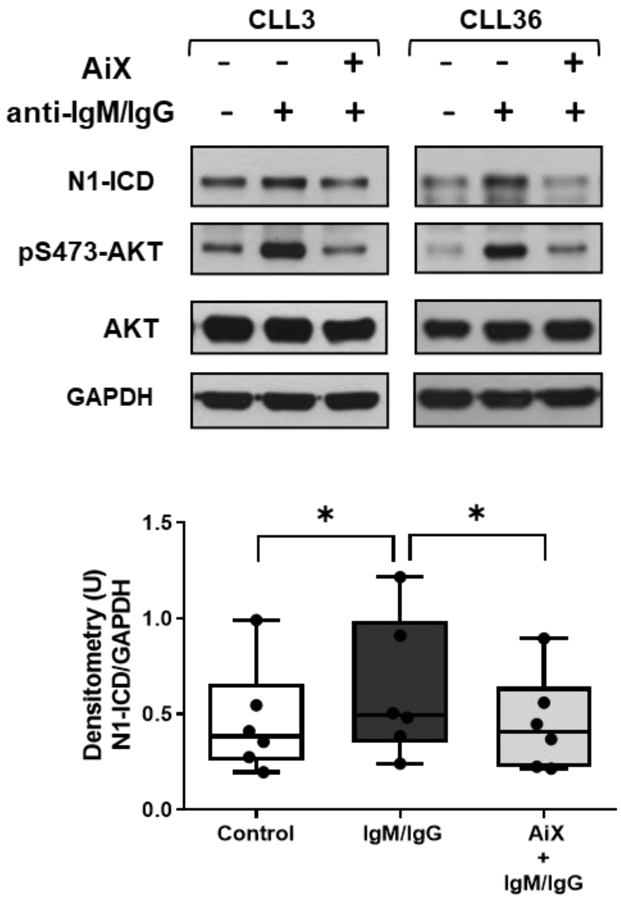

Supplementary Figure S5. Effect of AKT inhibition on BCR-induced N1-ICD levels in CLL cells. CLL cells from *IGHV*-unmutated patients were pretreated for 6 h with 5μM AKTiX (AiX) or complete medium as control, and then stimulated for 15 minutes with anti-IgM/IgG antibodies 10μg/ml (n=6). Top panel, expression of N1-ICD and GAPDH, as a loading control, was examined by Western blot analysis. AKT activation and inhibition by BCR stimulation or AiX treatment, respectively, was verified by analyzing AKT phosphorylation at Serine 473 (pS473-AKT). Two representative cases are shown. Bottom panel, box and whisker plots with data points of densitometry analysis of N1-ICD are shown. Densitometry units (U) were calculated relative to GAPDH. *$P < 0.05$ according to Wilcoxon paired test.

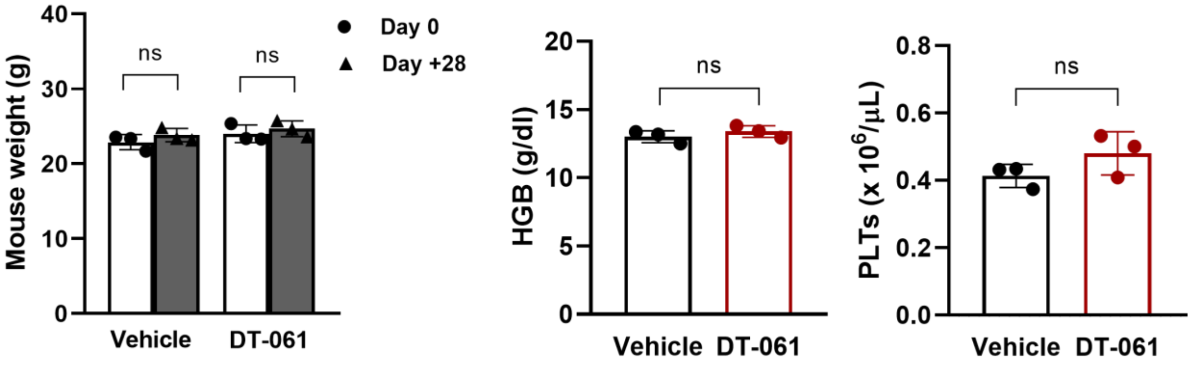

Supplementary Figure S6. Effect of DT-061 treatment on animal weight, hemoglobin levels and platelet count in the Eμ-TCL1 mouse model of CLL. The bar graphs with data points indicate the animal weight determined at the start (day 0) and the end (day +28) of treatment with DT-061 (n=3) or vehicle (n=3) in the left panel; and the hemoglobin levels (HGB, g/dl) and platelet count (PLT, x10^6/μl) determined at the end of the treatment (day +28) with DT-061- (n=3) or vehicle (n=3), in the middle and right panels, respectively. Data are presented as the mean ± SD of 3 mice per group. ns = not significant according to unpaired Student's *t* test.
