# Supplementary material for: GSK3β is a critical, druggable component of the network regulating the active NOTCH1 protein and cell viability in CLL
Source: Cell Death Dis. 2022 Sep 1;13(9):755. doi: 10.1038/s41419-022-05178-w (PMC9436923; doi:10.1038/s41419-022-05178-w)
Supplement: Supplementary file 3 — Change of Authorship Request [file 41419_2022_5178_MOESM3_ESM.pdf]

SPRINGER NATURE

Change of authorship request form - Journals (pre-acceptance)

Important information. Please read.

- This form should be used by authors to request any change in authorship (adding/deleting authors) including changes in corresponding authors. This form should not be used for name changes. Please fully complete all sections. Use black ink and block capitals and provide each author's full name with the given name first followed by the family name.

- By signing this declaration, all authors guarantee that the order of the authors are in accordance with their scientific contribution, if applicable as different conventions apply per discipline, and that only authors have been added who made a meaningful contribution to the work.

- Please note, in author collaborations where there is formal agreement for representing the collaboration, it is sufficient for the representative or legal guarantor (usually the corresponding author) to complete and sign the Authorship Change Form on behalf of all authors, **next to the added/removed author(s)**. **(Complete Section 3, followed by Section 6.)**
  In author collaborations where there is no formal agreement for representing the collaboration and **there are more than 10 authors**, one may sign for all, provided the signer appends correspondence that attests that each of the authors have agreed to the change **and the added/removed authors sign the form**. **(Complete Section 3, followed by Section 6.)**

- Please note, we cannot investigate or mediate any authorship disputes. If you are unable to obtain agreement from all authors (including those who you wish to be removed) you must refer the matter to your institution(s) for investigation. Please inform us if you need to do this.

- If you are not able to return a fully completed form within **30 days** of the date that it was sent to the author requesting the change, we may have to withdraw your manuscript. We cannot publish manuscripts where authorship has not been agreed by all authors (including those who have been removed).

- Incomplete forms will be rejected.

- Please return/upload this form, fully completed, to the Journals Editorial Office. The Journal and/or Publisher will consider the information you have provided to decide whether to approve the proposed change in authorship. We may decide to contact your institution for more information or undertake a further investigation, if appropriate, before making a final decision.

SPRINGER NATURE

Change of authorship request form - Journals (pre-acceptance)

Section 1: Please provide the current title of manuscript

Manuscript ID no.: CDDIS-22-0093R

Title: **GSK3β is a critical, druggable component of the network regulating the active NOTCH1 protein and cell viability in CLL**

Section 2: Please provide the previous authorship, in the order shown on the manuscript before the changes were introduced. Please indicate the corresponding author by adding (CA) behind the name.

	First name(s)	Family name	ORCID or SCOPUS id, if available
1st author	FILOMENA	DE FALCO	
2nd author	CHIARA	ROMPIETTI	
3rd author	DANIELE	SORCINI	
4th author	ANNARITA	SCIALDONE	
5th author	STEFANO	BALDONI	
6th author	BEATRICE	DEL PAPA	
7th author	FRANCESCO MARIA	ADAMO	
8th author	ESTEVÃO CARLOS	SILVA BARCELOS	
9th author	ERICA	DORILLO	
10th author	ARIANNA	STELLA	

Please use an additional sheet if there are more than 10 authors.

SPRINGER NATURE Springer Nature is one of the world's leading global research, educational and professional publishers, created in May 2015 through the combination of Nature Publishing Group, Palgrave Macmillan, Macmillan Education and Springer Science+Business Media.

SPRINGER NATURE

Change of authorship request form - Journals (pre-acceptance)

Section 1: Please provide the current title of manuscript

Manuscript ID no.: CDDIS-22-0093R

Title: **GSK3β is a critical, druggable component of the network regulating the active NOTCH1 protein and cell viability in CLL**

Section 2: Please provide the previous authorship, in the order shown on the manuscript before the changes were introduced. Please indicate the corresponding author by adding (CA) behind the name.

	First name(s)	Family name	ORCID or SCOPUS id, if available
11th ~~1st~~ author	MAURO	DI IANNI	
12th ~~2nd~~ author	ISABELLA	SCREPANTI	
13th ~~3rd~~ author	PAOLO	SPORTOLETTI (CA)	
14th ~~4th~~ author	EMANUELA	ROSATI (CA)	
~~5th author~~			
~~6th author~~			
~~7th author~~			
~~8th author~~			
~~9th author~~			
~~10th author~~			

Please use an additional sheet if there are more than 10 authors.

SPRINGER NATURE Springer Nature is one of the world's leading global research, educational and professional publishers, created in May 2015 through the combination of Nature Publishing Group, Palgrave Macmillan, Macmillan Education and Springer Science+Business Media.

Change of authorship request form - Journals (pre-acceptance)

Section 3: Please provide a justification for change. Please use this section to explain your reasons for changing the authorship of your manuscript, e.g. what necessitated the change in authorship? Please refer to the (journal) policy pages for more information about authorship. Please explain why omitted authors were not originally included and/or why authors were removed on the submitted manuscript.

IN THE REVISED VERSION OF THE MANUSCRIPT WE HAVE ADDED ANGELA ESPOSITO AS A NEW CO-AUTHOR, BECAUSE SHE CONTRIBUTED TO THE EXPERIMENTS REQUESTED FOR THE REVISION. IN PARTICULAR, SHE PERFORMED WESTERN BLOT ANALYSIS/DATA ACQUISTION INCLUDED IN ALL THE REVISED FIGURES. ANGELA ESPOSITO WAS NOT ORIGINALLY INCLUDED AS SHE DID NOT CONTRIBUTE TO THE EXPERIMENTS PRESENTED IN THE ORIGINALLY SUBMITTED MANUSCRIPT.

Section 4: Proposed new authorship. Please provide your new authorship list in the order you would like it to appear on the manuscript. Please indicate the corresponding author by adding (CA) behind the name. <u>If the Corresponding Author has changed, please indicate the reason under section 3</u>.

	First name(s)	Family name (this name will appear in full on the final publication and will be searchable in various abstract and indexing databases)	Affiliated institute	E-mail address
1st author	FILOMENA	DE FALCO	University of Perugia	defalcofil@libero.it
2nd author	CHIARA	ROMPIETTI	University of Perugia	rompiettic@yahoo.it
3rd author	DANIELE	SORCINI	University of Perugia	daniele.sorcini@unipg.it
4th author	ANGELA	ESPOSITO	University of Perugia	angela.esposito@unipg.it
5th author	ANNARITA	SCIALDONE	University of Perugia	annarita.scialdone@unipg.it
6th author	STEFANO	BALDONI	University of Perugia-University of Chieti-Pescara	stefano.baldoni@unich.it
7th author	BEATRICE	DEL PAPA	University of Perugia	beadel@libero.it
8th author	FRANCESCO MARIA	ADAMO	University of Perugia	francesco91adamo@gmail.com
9th author	ESTEVÃO CARLOS	SILVA BARCELOS	University of Perugia	estevaocarlosbarcelos@gmail.com
10th author	ERICA	DORILLO	University of Perugia	erica.dorillo@unipg.it

Please use an additional sheet if there are more than 10 authors.

SPRINGER NATURE

Change of authorship request form - Journals (pre-acceptance)

Section 3: Please provide a justification for change. Please use this section to explain your reasons for changing the authorship of your manuscript, e.g. what necessitated the change in authorship? Please refer to the (journal) policy pages for more information about authorship. Please explain why omitted authors were not originally included and/or why authors were removed on the submitted manuscript.

IN THE REVISED VERSION OF THE MANUSCRIPT WE HAVE ADDED ANGELA ESPOSITO AS A NEW CO-AUTHOR, BECAUSE SHE CONTRIBUTED TO THE EXPERIMENTS REQUESTED FOR THE REVISION. IN PARTICULAR, SHE PERFORMED WESTERN BLOT ANALYSIS/DATA ACQUISTION INCLUDED IN ALL THE REVISED FIGURES. ANGELA ESPOSITO WAS NOT ORIGINALLY INCLUDED AS SHE DID NOT CONTRIBUTE TO THE EXPERIMENTS PRESENTED IN THE ORIGINALLY SUBMITTED MANUSCRIPT.

Section 4: Proposed new authorship. Please provide your new authorship list in the order you would like it to appear on the manuscript. Please indicate the corresponding author by adding (CA) behind the name. <u>If the Corresponding Author has changed, please indicate the reason under section 3</u>.

	First name(s)	Family name (this name will appear in full on the final publication and will be searchable in various abstract and indexing databases)	Affiliated institute	E-mail address
11th ~~1st~~ author	ARIANNA	STELLA	University of Perugia	arianna.stella89@gmail.com
12th ~~2nd~~ author	MAURO	DI IANNI	University of Chieti-Pescara-Ospedale Civile "Santo Spirito", ASL Pescara	mauro.diianni@unich.it
13th ~~3rd~~ author	ISABELLA	SCREPANTI	University of Rome "La Sapienza"	isabella.screpanti@uniroma1.it
14th ~~4th~~ author	PAOLO	SPORTOLETTI (CA)	University of Perugia	paolo.sportoletti@unipg.it
15th ~~5th~~ author	EMANUELA	ROSATI (CA)	University of Perugia	emanuela.rosati@unipg.it
~~6th author~~				
~~7th author~~				
~~8th author~~				
~~9th author~~				
~~10th author~~				

Please use an additional sheet if there are more than 10 authors.

SPRINGER NATURE Springer Nature is one of the world's leading global research, educational and professional publishers, created in May 2015 through the combination of Nature Publishing Group, Palgrave Macmillan, Macmillan Education and Springer Science+Business Media.

Change of authorship request form - Journals (pre-acceptance)

Section 5: Author contribution, Acknowledgement and Disclosures. Please use this section to provide a new disclosure statement and, if appropriate, acknowledge any contributors who have been removed as authors and ensure you state what contribution any new authors made (if applicable per the journal or book (series) policy). Please ensure these are updated in your manuscript - after approval of the change(s) - as our production department will not transfer the information in this form to your manuscript.

New acknowledgements:

This work was funded by the Associazione Italiana per la Ricerca sul Cancro (AIRC) (IG 2018-ID. 21352 to PS); Italian Ministry of Education, University and Research (MIUR) (Scientific Independence of Young Researchers-SIR Project-ID. RBSI14GPBL to PS); FIRC-AIRC (three-year fellowship "Filomena Todini" ID. 23928 to CR).

New Disclosures (financial and non-financial interests, funding):

The authors declare no competing interests.

New Author Contributions statement (if applicable per the journal policy):

FDF designed and performed in vitro experimental research, interpreted data and performed statistical analyses and figures; CR performed in vivo studies; DS acquired and analyzed flow cytometric data; AE acquired and analyzed Western blot data; AS contributed to confocal microscopy analyses; SB, BDP, ED, AS contributed to molecular experiments; FMA and ECSB contributed to Western blot analyses; IS and MDI provided critical suggestions; ER and PS designed research, conceived the project and supervised the work-flow; ER wrote the manuscript. All authors read and approved the final paper.

State 'Not applicable' if there are no new authors.

Change of authorship request form - Journals (pre-acceptance)

Section 6: Declaration of agreement. *All* authors, unchanged, new and removed *must* sign this declaration.
(NB: Please print the form, (docu)-sign and return/upload a scanned copy. Please note that signatures that have been inserted as an image file are acceptable as long as it is handwritten. Typed names in the signature box are unacceptable.) *** Please delete as appropriate. Delete all of the bold if you were on the original authorship list and are remaining as an author.**

	First name	Family name		Signature	Date
1st author	FILOMENA	DE FALCO	I agree to the proposed new authorship shown in section 4	Filomena De Falco	27/6/22
2nd author	CHIARA	ROMPIETTI	I agree to the proposed new authorship shown in section 4	Chiara Rompietti	June 27, 2022
3rd author	DANIELE	SORCINI	I agree to the proposed new authorship shown in section 4	Daniele Sorcini	June 27, 2022
4th authors	ANGELA	ESPOSITO	I agree to the proposed new authorship shown in section 4 **and the addition of my name to the authorship list**	Angela Esposito	June 27, 2022
5th author	ANNARITA	SCIALDONE	I agree to the proposed new authorship shown in section 4	Annarita Scialdone	June 27, 2022
6th author	STEFANO	BALDONI	I agree to the proposed new authorship shown in section 4	Stefano Baldoni	June 24, 2022
7th author	BEATRICE	DEL PAPA	I agree to the proposed new authorship shown in section 4	Beatrice Del Papa	June 28, 2022

SPRINGER NATURE

Change of authorship request form - Journals (pre-acceptance)

Section 6: Declaration of agreement. *All* authors, unchanged, new and removed *must* sign this declaration.
(NB: Please print the form, (docu)-sign and return/upload a scanned copy. Please note that signatures that have been inserted as an image file are acceptable as long as it is handwritten. Typed names in the signature box are unacceptable.) *** Please delete as appropriate. Delete all of the bold if you were on the original authorship list and are remaining as an author.**

	First name	Family name		Signature	Date
8th ~~1st~~ author	FRANCESCO MARIA	ADAMO	I agree to the proposed new authorship shown in section 4	[illegible]	June 27, 2022
9th ~~2nd~~ author	ESTEVÃO CARLOS	SILVA BARCELOS	I agree to the proposed new authorship shown in section 4	[illegible]	June 28, 2022
10th ~~3rd~~ author	ERICA	DORILLO	I agree to the proposed new authorship shown in section 4	[illegible]	June 28, 2022
11th ~~4th~~ authors	ARIANNA	STELLA	I agree to the proposed new authorship shown in section 4	[illegible]	June 28, 2022
12th ~~5th~~ author	MAURO	DI IANNI	I agree to the proposed new authorship shown in section 4	[illegible]	June 23, 2022
13th ~~6th~~ author	ISABELLA	SCREPANTI	I agree to the proposed new authorship shown in section 4	[illegible]	June 16, 2022
14th 7th author	PAOLO	SPORTOLETTI	I agree to the proposed new authorship shown in section 4	[illegible]	June 27, 2022

SPRINGER NATURE Springer Nature is one of the world's leading global research, educational and professional publishers, created in May 2015 through the combination of Nature Publishing Group, Palgrave Macmillan, Macmillan Education and Springer Science+Business Media.

SPRINGER NATURE

Change of authorship request form - Journals (pre-acceptance)

	First name	Family name		Signature	Date
15th ~~8th~~ author	EMANUELA	ROSATI	I agree to the proposed new authorship shown in section 4	[signature]	June 27, 2022
~~9th author~~			I agree to the proposed new authorship shown in section 4 **/and the addition/removal*of my name to the authorship list** /and the proposed change in corresponding author		
~~10th author~~			I agree to the proposed new authorship shown in section 4 **/and the addition/removal*of my name to the authorship list** /and the proposed change in corresponding author		

Please use an additional sheet if there are more than 10 authors.

In case of author collaborations with formal agreement:

	Name of consortium/consortia	First name	Family name		Signature	Date
Representative/legal guarantor				I agree to the proposed new authorship shown in section 4 **/and the addition/removal*of my name to the authorship list** /and the proposed change in corresponding author		

Both added/removed authors should complete the information in the first table under Section 6.

---- End of form ----

SPRINGER NATURE Springer Nature is one of the world's leading global research, educational and professional publishers, created in May 2015 through the combination of Nature Publishing Group, Palgrave Macmillan, Macmillan Education and Springer Science+Business Media.
